# A comprehensive estimate of recent carbon sinks in China using both top-down and bottom-up approaches

**DOI:** 10.1038/srep22130

**Published:** 2016-02-29

**Authors:** Fei Jiang, Jing M. Chen, Lingxi Zhou, Weimin Ju, Huifang Zhang, Toshinobu Machida, Philippe Ciais, Wouter Peters, Hengmao Wang, Baozhang Chen, Lixin Liu, Chunhua Zhang, Hidekazu Matsueda, Yousuke Sawa

**Affiliations:** 1 Jiangsu Provincial Key Laboratory of Geographic Information Science and Technology, International Institute for Earth System Science, Nanjing University, Nanjing 210046, China; 2 Department of Geography, University of Toronto, Ontario M5S3G3, Canada; 3 Chinese Academy of Meteorological Sciences, China Meteorological Administration, Beijing 100081, China; 4 State Key Laboratory of Resources and Environment Information System, Institute of Geographic Sciences and Natural Resources Research, Chinese Academy of Sciences, Beijing 100101, China; 5 National Institute for Environmental Studies, 305-8506 Tsukuba, Japan; 6 Laboratoire des Sciences du Climat et de l’Environnement, CEA CNRS UVSQ, 91191 Gif sur Yvette, France; 7 Department of Meteorology and Air Quality, Wageningen University and Research Center, 6708 PB Wageningen, The Netherlands; 8 University of Groningen, Centre for Isotope Research, 9747 AG Groningen, The Netherlands; 9 Geochemical Research Department, Meteorological Research Institute, Tsukuba 305-0052, Japan; 10 School of Geography and Planning, Ludong University, Yantai 264025, China

## Abstract

Atmospheric inversions use measurements of atmospheric CO_2_ gradients to constrain regional surface fluxes. Current inversions indicate a net terrestrial CO_2_ sink in China between 0.16 and 0.35 PgC/yr. The uncertainty of these estimates is as large as the mean because the atmospheric network historically contained only one high altitude station in China. Here, we revisit the calculation of the terrestrial CO_2_ flux in China, excluding emissions from fossil fuel burning and cement production, by using two inversions with three new CO_2_ monitoring stations in China as well as aircraft observations over Asia. We estimate a net terrestrial CO_2_ uptake of 0.39–0.51 PgC/yr with a mean of 0.45 PgC/yr in 2006–2009. After considering the lateral transport of carbon in air and water and international trade, the annual mean carbon sink is adjusted to 0.35 PgC/yr. To evaluate this top-down estimate, we constructed an independent bottom-up estimate based on ecosystem data, and giving a net land sink of 0.33 PgC/yr. This demonstrates closure between the top-down and bottom-up estimates. Both top-down and bottom-up estimates give a higher carbon sink than previous estimates made for the 1980s and 1990s, suggesting a trend towards increased uptake by land ecosystems in China.

The carbon balance of China is characterized by the World’s highest emissions of CO_2_ from fossil fuel use and substantial carbon sequestration in intensively managed ecosystems. The large land area of China (6.4% of the global land mass), coupled to its rapid economic development, its large food production, and its recent large-scale afforestation practices puts its carbon cycle in the center of current global carbon cycle research. Top-down atmospheric inversions^1^,^2^ have used globally distributed stations measuring atmospheric CO_2_ mole fraction observations to provide estimates of surface-atmosphere CO_2_ fluxes over large (>10^6^ km^2^) spatial areas. One limitation of inversions is the insufficient density of atmospheric stations over continental regions. In this study, we derive new top-down calculations of China’s CO_2_ budget, by combining new atmospheric CO_2_ observations within and around China, with two independent atmospheric inversion systems. We additionally conduct a synthesis of the bottom-up carbon budget of China’s terrestrial ecosystems to gauge the convergence between these independent streams of information.

## Results

### Top-down estimate

Atmospheric inversions^3^, quantify net CO_2_ fluxes at the surface of the Earth, based on transport models and atmospheric CO_2_ observations. In this process, a higher density of observations allows more detailed estimates of fluxes. In China, only one high altitude monitoring station (Mt Waliguan) in the western part of China^4^ has been available to constrain the published estimates up until 2006. Since then, the Chinese Meteorological Administration (CMA) installed three additional surface GHG monitoring stations^5^. In addition, CO_2_ measurements on board of passenger aircraft, with vertical profiles at selected airport locations and horizontal transects at the cruising altitude of aircraft, have been acquired over Asia and Europe^6^ since July 2005 by the Comprehensive Observation Network for Trace gases by AirLiner project (CONTRAIL). These CO_2_ observations form the basis for the revised top-down estimate of the CO_2_ budget of China.

**Figure 1:**
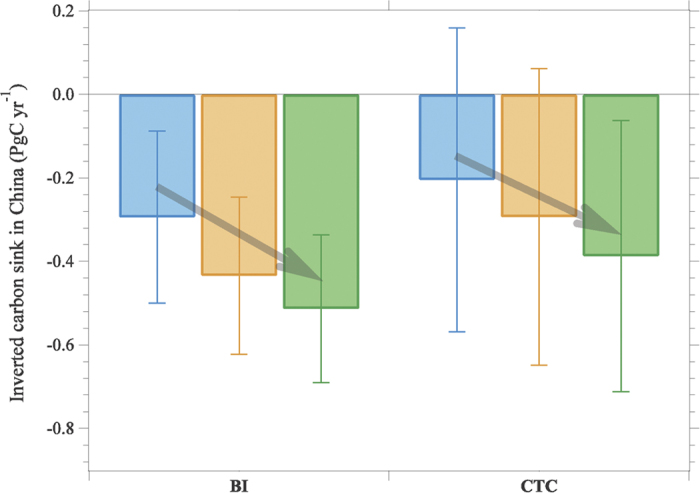
Inverted carbon sinks in China during 2006–2009 from two inversion systems. Bayesian Inversion (BI) and Carbon Tracker-China (CTC). Values have been adjusted with the national CO_2_ emissions from fossil fuel burning, cement manufacture, and gas flaring of 1.90 PgC yr^−1^ during 2006–2009 reported by the Carbon Dioxide Information Analysis Center^9^. Blue: constrained only with global CO_2_ datasets; orange: constrained with additional China Meteorological Administration (CMA)’s measurements (3 sites); and green: constrained with additional CMA and CONTRAIL aircraft CO_2_ measurements.

We use two well-established inversion systems, a nested Bayesian inversion (BI) system^7^ and the CarbonTracker-China (CTC) system^8^ to estimate CO_2_ fluxes in China during the 2000s. Details of both systems are provided in the Supplementary Material. Using the inversion systems with only the Mt Waliguan CO_2_ record as constraint, we estimate over China a net sink of atmospheric CO_2_ during 2006–2009, excluding CO_2_ emissions from fossil fuels and cement. The CO_2_ sink estimates are of 0.29 ± 0.21 and 0.20 ± 0.36 PgC yr^−1^(1-sigma posterior Gaussian uncertainties), respectively in each inversion. These mean values are close to previous inversion estimates, in the range of 0.16~0.35 PgC yr^−1^ during 1996–2009^1^,^9^,^10^,^11^,^12^. When the three new CMA stations are assimilated into the BI and CTC systems, the inverted terrestrial CO_2_ sink in China increases to 0.43 ± 0.19 and 0.29 ± 0.35 PgC yr^−1^, respectively. When both CMA and CONTRAIL data are assimilated, the sink further increases to 0.51 ± 0.18 and 0.39 ± 0.33 PgC yr^−1^, respectively (Fig. 1). The two inversion systems thus consistently show that when new CO_2_ measurements within or around China are included, the inverted CO_2_ sink in China gets larger and its uncertainty is reduced. With the new CO_2_ data added, the mean inverted CO_2_ sink in China is 0.45 ± 0.25 PgC yr^−1^, which is a higher than previous inversions. In inversions, the inferred sinks depend on the value being assumed for CO_2_ emissions from fossil fuel burning and cement production (FFCO_2_). There is a rather large uncertainty of FFCO_2_ in China, as evidenced by differences between published estimates^13^,^14^. In this study, we used as a reference FFCO_2_ from the Carbon Dioxide Information Analysis Center (CDIAC)^15^, of 1.90 PgC yr^−1^ during 2006–2009 (Table 1). Using the value of FFCO_2_ recently produced by Liu *et al*.^14^, based on a downward revision of the carbon content of coal burned in China, which is 9% lower than CDIAC, would lead to a mean inverted CO_2_ sink in China of 0.28 ± 0.25 PgC yr^−1^.

### Bottom-up estimate

The top-down estimates of the carbon sources and sinks excluding FFCO_2_ should equal the change in carbon stocks in the various reservoirs involved in carbon exchange. Various methods, often referred to as bottom-up, have been developed to estimate these carbon stock changes. In order to evaluate specifically the new top-down estimate of China’s terrestrial ecosystems, we updated bottom-up carbon exchange estimate to cover the period of 2006–2009.

We reconstructed carbon stock changes of vegetation and soil in China during 2000s (Table 2). For vegetation carbon stock change, forest is the most important biome. Based on the 6^th^ (1999–2003) and 7^th^ (2004–2008) national forest inventories, Zhang *et al*.^16^, Guo *et al*.^17^ and Pan *et al*.^18^ estimated that forest biomass carbon stock accumulated at a rate of 0.174, 0.104, and 0.115 PgC yr^−1^ during the 2000s, respectively. We use the mean and standard deviation of these three estimates, which is 0.13 ± 0.038 PgC yr^−1^. This is larger than the value of 0.075 ± 0.035 PgC yr^−1^ reported by Piao *et al*.^9^ during the 1980s and 1990s, suggesting that forest biomass carbon gains in 2000s significantly increased from 1980s and 1990s. In addition, short rotation forests and bamboo plantations were estimated to have accumulated 0.009 ± 0.006 PgC yr^−1^ in total^16^,^17^, and woodlands, shrub, tree on non-forest lands were estimated to have a sink of 0.016 ± 0.011 PgC yr^−1^ in total^16^. Due to lack of more recent research results for grasslands, we use the same estimate than Piao *et al*.^9^ of 0.007 ± 0.003 PgC yr^−1^. In total, vegetation biomass in China accumulated 0.17 ± 0.060 PgC yr^−1^ of carbon during 2000s.

For soil carbon stock (SOC) change, we first estimate the rate of change of forest soil carbon stock, include dead wood, litter and soil carbon, to be 0.068 ± 0.034 PgC yr^−1^ during 2000s using the InTEC model^19^. This estimate is consistent with the value of 0.060 ± 0.030 PgC yr^−1^ for 2000–2007 estimated using ratios of soil carbon to vegetation biomass by Pan *et al*.^18^, but much higher than the value of 0.004 ± 0.015 PgC yr^−1^ in the statistical models of Piao *et al*.^9^ for 1996–2005. Since Piao’s statistical models were only able to explain 23~29% of the observed forest lands soil carbon variations, and their result is one order of magnitude lower than those of this study and Pan *et al*.^18^, we do not adopt Piao’s result in this study. We use the midpoint of the InTEC model and Pan *et al*.^18^ of 0.064 ± 0.030 PgC yr^−1^ as the rate of SOC accumulation in Chinese forests. For shrub lands, Piao *et al*.^9^ estimated the changes of SOC, using a statistical model, to be 0.039 ± 0.009 PgC yr^−1^ during 1982–1999, and using a process model, Tian *et al*.^20^ reported that shrub SOC accumulated an average 0.012 ± 0.005 PgC yr^−1^ from 1981 to 2001. We use the average of these two studies of 0.026 ± 0.019 PgC yr^−1^ for this biome. For cropland and grassland SOC, we directly use the estimates of 0.021 ± 0.004 PgC yr^−1^ and 0.005 ± 0.002 PgC yr^−1^, respectively, reported by Huang *et al*.^21^. In Total, this gives a bottom-up SOC accumulation rate of 0.12 ± 0.060 PgC yr^−1^ during the 2000s. Therefore, the bottom-up estimate is a net carbon accumulation in land ecosystems of 0.29 ± 0.12 PgC yr^−1^ in China.

### Consistent top-down and bottom-up estimates

The bottom-up estimate of carbon stock change in vegetation and soil is still much lower than the inversion results. That is because inland waters, ocean and wood products are also reservoirs for terrestrial carbon and the inverted CO_2_ sink is also influenced by CO_2_ from the oxidization of net imported products and reduced carbon compounds (RCC) emitted from fossil fuels and ecosystems. We then try to reconcile top-down and bottom-up results as follows:


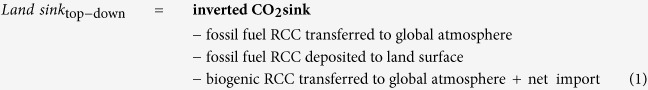



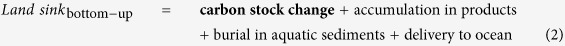



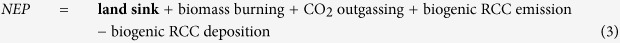


where biogenic RCC includes RCC from biomass burning and biogenic volatile organic carbon emissions. Net ecosystem productivity (NEP) is the difference between photosynthesis and respiration in terrestrial ecosystems, which is also estimated from the top-down and bottom-up results to provide a full picture of the carbon cycle in China. All items in the above equations are positive. Equations (2) and (3) are straightforward to understand, and details of equation (1) are given in the Supplementary Material.

Fossil fuel emission inventories, i.e., CDIAC, are based on CO_2_ emission factors that include direct emissions of CO_2_ from fossil fuels and emissions of RCC, e.g., carbon monoxide (CO), methane (CH_4_) and non-methane volatile organic carbons (NMVOCs) that are later oxidized into CO_2_^22^. When the total fossil fuel emission is treated as all CO_2_ emission, as done in most inversion studies including ours, the contribution of the emission to the regional near surface CO_2_ concentration is overestimated. That is because, after emission to the atmosphere, NMVOCs is first oxidized to CO, which is subsequently oxidized to CO_2_. The NMVOCs oxidation process is typically fast (hours), while the CO oxidation process is rather slow (1–2 months). CH_4_ is also oxidized to CO_2_ at a very slow rate. Generally, these oxidation processes will occur during the air mass transport, and therefore non-CO_2_ carbon species emitted from one region (e.g., China) will transform into CO_2_ globally rather than locally. Hence, the treatment of non-CO_2_ carbon species as direct CO_2_ emission in inversions will tend to overestimate the contribution of fossil fuel emission to CO_2_ concentration over China in inversions, causing overestimation of the inverted carbon sink, i.e. too high sinks needed to offset CO_2_ gradients due to too high emissions^23^. During 2006–2009, China emitted RCC at a rate of 0.102 ± 0.007 PgC yr^−1^, including 0.072 ± 0.005 PgC yr^−1^of CO, 0.019 ± 0.001 PgC yr^−1^of NMVOCs and 0.011 ± 0.001 PgC yr^−1^ of CH_4_ on average, roughly 14% of these emissions were converted to CO_2_ in the boundary layer over China, 12% were deposited to the land surface, and the remaining 74% were transported to the global atmosphere^24^. Therefore, the “fossil fuel RCC transferred to global atmosphere” term in Eq. 1 is 0.076 ± 0.0050 PgC yr^−1^, and the “fossil fuel RCC deposited to land” term in Eq. 1 is 0.012 ± 0.0010 PgC yr^−1^.

The Global Fire Emission Database (GFED) biomass burning emission dataset, explicitly separates CO_2_, CO, CH_4_ and NMVOC emissions. Based on GFED v3.1^25^, emissions of CO_2_ and RCC from biomass burning are 0.016 and 0.0020 PgC yr^−1^, respectively over China. In addition, land ecosystems also directly release biogenic RCC, including NMVOC and CH_4_. Their emissions over China are estimated to be 0.021 ± 0.010 and 0.027 ± 0.013 PgC yr^−1^, respectively. The “biogenic RCC emission” term in Eq. 3 is 0.050 ± 0.024 PgC yr^−1^, and the “biogenic RCC deposition” term in Eq. 3 is 0.0060 ± 0.0020 PgC yr^−1^, taken as 12% of the sum. The “biogenic RCC transferred to global atmosphere” term in Eq. 1 is 0.037 ± 0.018 PgC yr^−1^, which is 74% of the biogenic RCC emission. It is a negative adjustment to the top-down land sink estimate because 74% of the biogenic RCC (carbon source) is lost to the global atmosphere and not captured by the inversion^24^.

The net imports of forest and crop products from outside China, which are decomposed in China and become additional sources of carbon to the atmosphere are included in the top-down sink estimates, but should be subtracted from it to be compared with the bottom-up ecosystem carbon stock change estimate, which does not count wood and crop products stocks. Moreover, carbon accumulated in forest products is a net accumulation of carbon that should be included in the bottom-up estimate^26^. Based on the Food and Agriculture Organization of the United Nations (FAO) statistical databases^27^, we estimate that 0.012 PgC of wood and 0.019 PgC of food were imported into China every year during 2000s. The net imported food is assumed to be fully consumed and oxidized to CO_2_ in the same year, while only a portion of the net imported wood products is fully oxidized. The remainder goes into long-term products and is slowly oxidized over time. Using the method of Winjum *et al*.^28^, we calculate that during 2006–2009, the CO_2_ emission due to net wood import is 0.006 PgC yr^−1^, and the net accumulation of carbon in wood products made by local harvests in China during 2000s is 0.005 PgC yr^−1^. Therefore, the “net import” term in Eq. 1 is 0.025 PgC yr^−1^, and the “accumulation in products” term in Eq. 2 is 0.0050 PgC yr^−1^.

Inland aquatic systems are now considered as a significant component of land-atmosphere CO_2_ fluxes^29^,^30^,^31^. Globally, about 2.1 PgC yr^−1^ of carbon are transported from terrestrial landscape to inland waters, in which 1.7 PgC yr^−1^ is from soil erosion and 0.4 PgC yr^−1^ is from rock weathering^32^. In this study, we estimate that inland waters of China annually receive 0.12 ± 0.06 PgC yr^−1^ of carbon from land, in which 0.105 ± 0.052 PgC yr^−1^ is from eroded soils and 0.015 ± 0.008 PgC yr^−1^ is from rock weathering (half of the exported dissolved inorganic carbon (DIC)^24^). During the inland water carbon transport processes, about 0.020 ± 0.010 PgC yr^−1^ of carbon is buried in aquatic sediments, and 0.062 ± 0.030 PgC yr^−1^ is returned to the atmosphere, and the remainder of 0.038 ± 0.019 PgC yr^−1^ is delivered to the coastal ocean, including 0.008 ± 0.004 PgC yr^−1^ of total organic carbon (TOC) and 0.030 ± 0.015 PgC yr^−1^ of DIC. The amounts of carbon that are transported to ocean, buried in the sediment and outgassed to the atmosphere in China are estimated to be all lower than those in Europe, but the relative fractions among burial, outgassing, and transport to the ocean are close to those in Europe^24^. Therefore, the “burial in aquatic sediments” term in Eq. 2 is 0.020 ± 0.010 PgC yr^−1^, the “delivery to ocean” term in Eq. 2 is 0.023 ± 0.010 PgC yr^−1^ (i.e., TOC + DIC/2), and the “CO_2_ outgassing” term in Eq. 3 is 0.062 ± 0.030 PgC yr^−1^.

**Figure 2:**
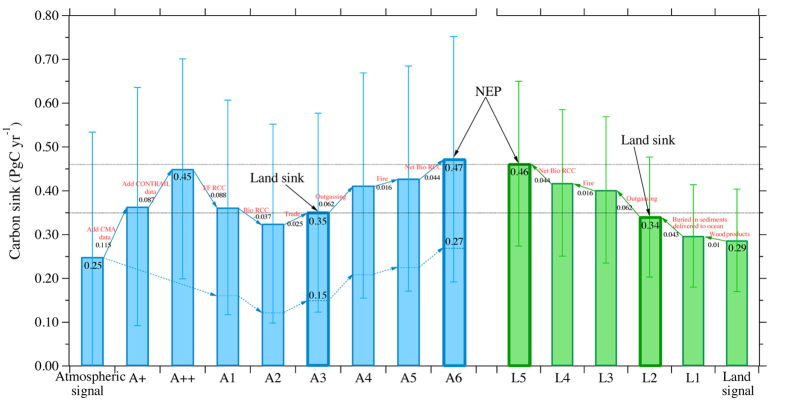
Terrestrial carbon balance in China. Atmospheric signal A: inversion result (exclude CO_2_ emissions from fossil fuels) constrained with global CO_2_ datasets only; A+: result of additional constraint with China Meteorological Administration (CMA)’s measurements (3 sites); A++: result of further constraint with CONTRAIL aircraft CO_2_ measurements; A1: result after considering the fossil fuels RCC emission and transformation; A2: result after correcting for the biogenic RCC lateral transport; A3: result after correcting for the net import through international trade. Land signal: carbon accumulated in China’s ecosystems; L1, result after considering the accumulation in harvested wood products; L2: result after considering the carbon burial and transport to ocean; A4, L3: result after considering the CO_2_ outgassing from inland waters; A5, L4: result after correcting for the biomass burning CO_2_ emission; A6, L5: result after correcting for the net RCC emission from ecosystems. The lower dotted blue curve indicates adjustments made to atmospheric inversion results without the added data. From land sink to NEP estimates, there are three major adjustments.

By including the various fluxes outlined above (see details in the Supplementary Material), the land sink estimates by the top-down and bottom-up methods are adjusted to 0.35 ± 0.23 (mean range 0.29–0.41) PgC yr^−1^ and 0.33 ± 0.14 PgC yr^−1^, respectively, and the corresponding NEP estimates are 0.47 ± 0.28 PgC yr^−1^ and 0.45 ± 0.19 PgC yr^−1^ (Fig. 2).

## Discussion

**Figure 3:**
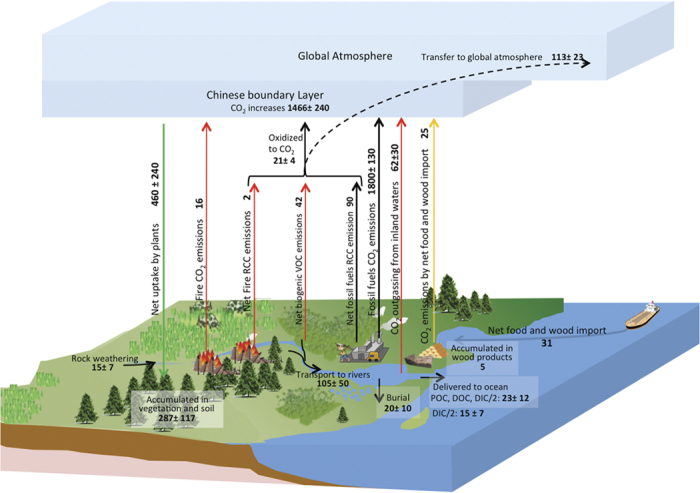
Carbon budgets of China’s terrestrial ecosystems from 2006 to 2009. Unit: TgC yr^−1^. The net CO_2_ flux in the boundary layer of China is 1.47 ± 0.24 PgC yr^−1^, which is the balance of 1.80 ± 0.13 PgC yr^−1^ directly emitted by fossil fuels and cement production, 0.016 PgC yr^−1^ directly emitted by biomass burning, 0.021 ± 0.004 PgC yr^−1^ converted from RCC which are emitted by fossil fuels, biomass burning and vegetation, 0.025 PgC yr^−1^ released by the consumption of food and wood imported from outside China, 0.062 ± 0.030 PgC yr^−1^ degassed from inland freshwaters, and about 0.46 ± 0.24 PgC yr^−1^ as the net uptake by terrestrial ecosystems. Out of the net uptake, about 0.29 ± 0.12 PgC yr^−1^ is accumulated in these ecosystems, 0.005 PgC yr^−1^ is accumulated in the harvested wood products, 0.105 ± 0.050 PgC yr^−1^ is transported to inland waters, 0.016 PgC yr^−1^ is emitted due to biomass burning, and 0.044 ± 0.020 PgC yr^−1^ is net released in the form of RCC. This figure was drew by F. Jiang.

The top-down and bottom-up estimates are consistent within their respective uncertainties. A full picture of the carbon cycle of China is shown in Fig. 3. However, considerable sources of systematic uncertainties still exist in these estimates, and the real uncertainties of both top-down and bottom-up estimates are likely higher than those calculated above.

The top-down results for south and southwest China are very uncertain (Supplementary Fig. S3, Fig. S4), although the results for eastern and northern China from different inversion systems are consistent within their uncertainties. Generally, significant and spatially explicit constraints on fluxes can be obtained in locations near and immediately upwind of surface measurements^33^. In south and southwest China there are no local surface CO_2_ observations and very few air masses from these regions move to existing observation stations (Supplementary Fig. S5). Although the inverted carbon sinks are significantly sensitive to the additional CO_2_ observations, the total error reduction is very limited, only about 10~14%. New atmospheric CO_2_ measurements in south, southwest, and central China should be added to improve this further.

We also assume that the fossil fuel emissions from China are perfectly known, and therefore fixed in the inversions, but previous studies show that there is an uncertainty of about 7~9% in these emissions^13^,^14^. This amounts to ~0.12 PgC yr^−1^ during 2006–2009. The systematic error of emissions is thus comparable to the random uncertainty of inversion results. However, since a bias in fossil fuel estimation would influence all inversions in the same way, our finding that the inverted carbon sink in China increases when the new CO_2_ observations used for China (as shown in Fig. 1) would not change if we adopted another fossil fuel estimate.

In the bottom-up approach, some estimates are very coarse and some are not included: 1) the conversion rate of 14% from non-CO_2_ species to CO_2_ in the boundary layer is from a simulation in Europe^24^, which may depend on air pollutants emission strength and the size of the region, and thus this value may be different for China’s landmass; 2) the carbon accumulation for harvested wood is estimated based on empirical coefficients of limited cases^28^; 3) carbon transport in inland waters is estimated based on limited measurements in main rivers and lakes of China, which do not cover the entire country, and the estimate of carbon transport from terrestrial ecosystem to rivers (0.105 ± 0.050 PgC yr^−1^) is lower than a recent result of 0.19~0.24 PgC yr^−1^ which was calculated using the Revised Universal Soil Loss Equation (RUSLE) model^34^; 4) a small amount of forest and shrub soil carbon may contribute to the lateral transport of carbon in rivers but this amount is not included in models used for these ecosystems, and therefore modeled soil carbon sinks may be overestimated by this small amount; and 5) emissions from the net import of meat and cooking oil and domestic biofuel consumption are not considered. Furthermore, the top-down estimate is for the late 2000s (2006–2009), while the bottom-up estimate is mainly for the 2000s. Recent evidence suggests that warmer temperatures in China since then^7^, as well as afforestation/reforestation of previously cleared land, has lead to an intensification of Asia’s land carbon sink that contributes partly to the increasing trend for the global land sink during 2000s^35^.

We conclude that the land sink in China’s terrestrial ecosystems is 0.34 ± 0.19 PgC yr^−1^ during 2000s, which is larger than the comprehensive estimate of 0.19~0.26 PgC yr^−1^ by Piao *et al*.^9^ for the 1980s and 1990s. In Piao’s estimate, burial in aquatic sediments and delivery to the oceans were not included. But it is also possible that the CO_2_ sink in China actually has intensified between the 1990s and the 2000s as other studies found that between 1989–1998 and 1999–2008, China’s forest area and carbon density increased by 14% and 12%, respectively, causing the biomass carbon sink to increase by 0.14 PgC yr^−1 ^16,18. Our results show that the use of additional CO_2_ observations within and around China doubles our top-down sink estimates and makes it possible to achieve the closure between top-down and bottom-up estimates.

## Materials and Methods

### CO_2_ observations

In the BI system, 130 sites from GLOBALVIEW-CO_2_ 2010 are used, and in the CTC system, 95 time series from the Observation Package data products (obspack v1.02) and 4 stations from the World Data Centre for Greenhouse Gases (WDCGG) are included. Weekly flask CO_2_ measurements from Jul 2006 to Dec 2009 at 3 sites operated by Chinese Academy of Meteorological Sciences, China Meteorological Administration (CAMS/CMA)^5^, and aircraft CO_2_ measurements from Nov 2005 to Dec 2009 over Eurasian by the Comprehensive Observation Network for Trace gases by AirLiner (CONTRAIL) project^6^ are used in both systems. The three CAMS/CMA sites are all regional background stations, which are located in Northeast China (LFS), North China (SDZ), and East China (LAN), and with altitudes of 330, 293 and 139 m, respectively. The air intake height is 10 m above ground level for all three sites. The measurements in these stations are sampled and analyzed using the recommended methods of WMO/GAW, and the accuracy is comparable with that of NOAA/ESRL^5^.

### Simulation for the soil carbon fluxes over forest land

The Integrated Terrestrial Ecosystem C-budget (InTEC) model^19^, which is a regional C-budget model, is used to simulate the soil carbon fluxes over forest land. It combines the CENTURY model for soil C and nutrient dynamics and Farquahar’s leaf biochemical model for canopy-level annual photosynthesis implemented using a temporal and spatial scaling scheme. In this study, the InTEC model is run from 1901 to 2012. The simulation region covers the whole China, with a horizontal resolution of 1 km × 1 km. LAI, NPP, forest cover and stand age data in 2005; climate data during 1901–2012, nitrogen deposition data during 1901–2010, soil data, and CO_2_ data during 1901–2012 were used to driving the InTEC model. No forest management was considered. Forest disturbance was considered according to the stand age. The simulation results for 2006 to 2009 are used in this study.

### RCC emissions in China

The Asian anthropogenic emission inventory for 2006 for the NASA INTEX-B Mission, and the Multi-resolution Emission Inventory for China (MEIC) for 2008 and 2010 are used to calculate the fossil fuel RCC emissions in China. The Global Fire Emission Data (GFED) V3.1 is used to calculate the biomass burning RCC emissions. The biogenic NMVOCs emissions are adopted from literature review. The CH_4_ emissions is from a top-down estimate by Klinger *et al*.^36^.

### Carbon transport by rivers

The carbon delivered to the ocean through rivers include dissolve organic carbon (DOC), dissolve inorganic carbon (DIC) and particulate organic carbon (POC). Nine Chinese exorheic rivers are considered, including the Yangtze River, Yellow River, Pearl River, Huai River, Hai River, Liao River, Songhua River, Qiantang River and Min River. For the Yangtze River, Yellow River and Pearl River, we use the observations by respectively Wu *et al*.^37^, Ran *et al*.^38^, and Zhang *et al*.^39^. For the Hai and Liao rivers, we use the observations by Xia and Zhang^40^, and for the other 4 rivers there are no observations available to date. We use several simple methods to estimate the transport: for DOC, we use the mean concentration of Yellow and Yangtze River, for DIC, we use the mean DIC/DOC ratio observed in the five rivers, and for POC we use an empirical formula^41^.

The CO_2_ outgassing from inland waters in China is calculated based on limited observations of CO_2_ outgassing rates in the past decade and the water surface area is reported by the National Bureau of Statistics in China. For rivers and steams, the observations in Pearl River, Yangtze River and Yellow River are used^42^,^43^,^44^; for reservoirs, the observations in five reservoirs in Yangtze River are used^45^; and for natural lakes, the average of global natural lakes^46^ is adopted directly.

The carbon burial in lakes and reservoirs are estimated using the data reported by Gui *et al*.^47^ and Dong *et al*.^48^, which both covered lakes in the middle and lower reaches of the Yangtze River Basin. The mean rate of these two studies is about two times the global mean rate. For the reservoirs, due to lack of observations, we assume that the carbon burial rate in Chinese reservoirs is also about two times of the global mean rate.

### Trade of food and wood

The import and export data of food and wood products from the FAO statistical databases^27^ are used. The food products include cereals, roots, sugar, soybeans and pulses, oil crops, vegetables, fruits, coffee and teas. The wood products include sawn wood, wood-based panels, paper and paperboard, recovered paper, other industry roundwood, and wood fuel and charcoal. Every year, the food products and the wood products of wood fuel and charcoal are assumed to be totally consumed and oxidized to CO_2_, while the other wood products are partially oxidized and partially go into uses or long-term storage, the CO_2_ release by these products are calculated using the method of Winjum *et al*.^28^.

### Carbon accumulated in wood products

This carbon is calculated using the local production data reported in the FAO statistical databases and the method of Winjum *et al*.^28^.

## Additional Information

**How to cite this article**: Jiang, F. *et al*. A comprehensive estimate of recent carbon sinks in China using both top-down and bottom-up approaches. *Sci. Rep.* **6**, 22130; doi: 10.1038/srep22130 (2016).

## Supplementary Material

Supplementary Information

## Figures and Tables

**Table 1:** Prior, optimized, and adjusted carbon flux from the inversion systems in China (PgC yr^−^ 1) for the period 2006–2009 (positive values represent carbon source, negative values represent carbon sink).

	BI	CTC
Prior bio flux	−0.10 ± 0.26	−0.092 ± 0.49
Fire emission	0.010	0.022
Fossil fuel emission	1.94	2.01
Optimized bio flux (Case 1)^1^	−0.34 ± 0.21	−0.33 ± 0.36
Optimized bio flux (Case_2)^1^	−0.48 ± 0.19	−0.42 ± 0.35
Optimized bio flux (Case_3)^1^	−0.56 ± 0.18	−0.51 ± 0.33
CDIAC	1.90	1.90
Adjusted bio flux (Case_1)^2^	−0.29 ± 0.21	−0.20 ± 0.36
Adjusted bio flux (Case_2)^2^	−0.44 ± 0.19	−0.29 ± 0.35
Adjusted bio flux (Case_3)^2^	−0.51 ± 0.18	−0.39 ± 0.33

Case_1: inversion result constrained with global CO_2_ datasets only; Case_2: result of additional constraint with China Meteorological Administration (CMA)’s measurements (3 sites); Case_3: result of further constraint with CONTRAIL aircraft CO_2_ measurements.

^1^inverted using inversion systems, and exclude fossil fuel and biomass burning CO_2_ emissions.

^2^further adjusted with the national CO_2_ emission reported in CDIAC, only exclude fossil fuel CO_2_ emissions (Adjusted bio flux = Fossil fuel emission + Fire emission + Optimized bio flux – CDIAC).

**Table 2:** Carbon accumulated in China’s terrestrial ecosystems during 2000s.

Category		Method	Area(1.0e6 ha)	Carbon balance (PgC yr^−1^)	Period	Ref.
Vegetation	Forest stands	Inventory	149	0.174	1999–2008	16
		Inventory	156	0.115	2000–2007	18
		Inventory	149	0.104	1999–2008	17
	**Forest ave.**		**151**	**0.13** ± **0.04**		
	Economic forests	Inventory	21	0.00	1999–2008	16
		Inventory	21	0.00	1999–2008	17
	**Economic Forest ave.**		**21**	**0.00**		
	Bamboo	Inventory	5.1	0.013	1999–2008	16
		Inventory	5.1	0.005	1999–2008	17
	**Bamboo ave.**	**Inventory**	**5.1**	**0.009** ± **0.006**		
	**Woodlands**	**Inventory**	**5.4**	**−0.002** ± **0.001**	1999–2008	16
	**Shrub**	**Inventory**	**49.5**	**0.019** ± **0.013**	1999–2008	16
	**Tree on non-forest lands**			**−0.001** ± **0.001**	1999–2008	16
	**Grass**	**Inventory**	**331**	**0.007** ± **0.003**	1980s,1990s	9
	**Subtotal**			**0.17** ± **0.060**		
Soil	Forest	InTEC model	155	0.068 ± 0.034	1999–2008	This study
		Inventory	156	0.060 ± 0.030	2000–2007	18
	**Forest ave.**		**155**	**0.064** ± **0.030**		
	Shrub	Statistic model	215	0.039 ± 0.009	1980s,1990s	9
		Process model	141	0.012 ± 0.005	1981–2000	20
	**Shrub ave.**			**0.026** ± **0.019**		
	**Crop**	**Aggregate**	**130**	**0.021** ± **0.004**	1980s,1990s	21
	**Grass**	**Aggregate**	**331**	**0.005** ± **0.002**	1980s,1990s	21
	**Subtotal**			**0.12** ± **0.060**		
	**Total**			**0.29** ± **0.12**		
