# Supplementary material for: A comprehensive estimate of recent carbon sinks in China using both top-down and bottom-up approaches
Source: Sci Rep. 2016 Feb 29;6:22130. doi: 10.1038/srep22130 (PMC4770414; doi:10.1038/srep22130)
Supplement: Supplementary Information [file srep22130-s1.doc]

**A comprehensive estimate of recent carbon sinks in China using both top-down and bottom-up approaches**

***Supplementary Information***

Fei Jiang^1^, Jing M. Chen^2^, Lingxi Zhou^3^, Weimin Ju^1^, Huifang Zhang^4^, Toshinobu Machida^5^, Philippe Ciais^6^, Wouter Peters^7,8^, Hengmao Wang^1^, Baozhang Chen^4^, Lixin Liu^3^, Chunhua Zhang^1,10^, Hidekazu Matsueda^9^, & Yousuke Sawa^9^

*^1^Jiangsu Provincial Key Laboratory of Geographic Information Science and Technology, International Institute for Earth System Science, Nanjing University, Nanjing 210046, China. ^2^Department of Geography, University of Toronto, Ontario M5S3G3, Canada.^3^Chinese Academy of Meteorological Sciences, China Meteorological Administration, Beijing 100081, China. ^4^State Key Laboratory of Resources and Environment Information System, Institute of Geographic Sciences and Natural Resources Research, Chinese Academy of Sciences, Beijing 100101, China. ^5^National Institute for Environmental Studies,* *305-8506 Tsukuba, Japan. ^6^Laboratoire des Sciences du Climat et de l'Environnement, CEA CNRS UVSQ, 91191 Gif sur Yvette, France. ^7^Department of Meteorology and Air Quality, Wageningen University and Research Center, 6708 PB Wageningen, The Netherlands. ^8^University of Groningen, Centre for Isotope Research, 9747 AG Groningen, The Netherlands. ^9^Geochemical Research Department, Meteorological Research Institute, Tsukuba 305-0052, Japan. ^10^School of Geography and Planning, Ludong University, Yantai 264025, China*

**S1. Inverse models**

**S1.1 Methods**

***1) Bayesian Inverse (BI) system***

We establish a nested atmospheric inversion system with a focus on China using the time-dependent Bayesian synthesis method ^1^. The key of this method is to minimize the following cost function ^1^.

$J=\frac{\mathbf{1}}{\mathbf{2}}\mathbf{(Ms-c)}^{\mathbf{T}}\mathbf{R}^{\mathbf{-1}}\mathbf{(Ms-c)+}\frac{\mathbf{1}}{\mathbf{2}}\mathbf{(s-s}_{\mathbf{p}}\mathbf{)}^{\mathbf{T}}\mathbf{Q}^{\mathbf{-1}}\mathbf{(s-s}_{\mathbf{p}}\mathbf{)}$ (1)

where **M** is a matrix representing the transport operator; **c** is the observations; **s** is the unknown vector of the carbon flux of all regions at different times combined with the initial well-mixed atmospheric CO_2_ concentration; **s_p_** is a priori estimation of **s**; and **R** and **Q** are the uncertainties of **c** and **s_p_**, respectively. By minimizing this cost function, the posterior fluxes ***s****_post_* and their uncertainties **Q***_post_* could be obtained as:

$\text{s}_{post}=\left(\text{M}^{\mathbf{T}}\mathbf{R}^{-\mathbf{1}}\text{M}+\mathbf{Q}^{-1}\right)^{-1}\left(\text{M}^{\mathbf{T}}\mathbf{R}^{-\mathbf{1}}\mathbf{c}+\mathbf{Q}^{-\mathbf{1}}\mathbf{s}_{\mathbf{p}}\right)$ (2)

$\text{Q}_{post}=\text{(Q}^{\text{-1}}+\text{M}^{\text{T}}\text{R}^{\text{-1}}\text{M)}^{\text{-1}}$ (3)

The global surface is separated into 43 regions based on the 22 TransCom large regions ^2^, with 13 small regions in China (Figure S1).The partition scheme for the 13 small regions is mainly based on land cover types, i.e. forest (5 regions), crop (4 regions), grass (3 regions), and desert (1 region). Monthly transport operators for the 43 regions are calculated using the global two-way nested transport model TM5 ^3^, which has been widely used in previous atmospheric inversion research ^4, 5^. In this study, TM5 is run at a horizontal resolution of 3˚ × 2˚ around the world without nesting a high-resolution domain, and a vertical structure of 25 layers, with the model top at about 1 hPa. TM5 was driven by offline meteorological fields taken from the European Centre for Medium-Range Weather Forecast (ECMWF) model. The meteorological fields have a 1˚ × 1˚ horizontal resolution, 60 vertical layers and 3 hours intervals for most variables.

Fossil fuel and biomass burning emissions are assumed to be perfectly known, and their contributions to the CO_2_ concentrations are pre-subtracted in the inversion system. These contributions are also simulated using TM5 with the same grid system as transport operator calculations. In this study, both fossil fuel and biomass burning emissions are obtained from the product of CarbonTracker CT2011_oi ^6^. The fossil fuel emission is named as the “Miller” emissions dataset, which is derived from independent global total and spatially-resolved inventories. Annual global total emissions are from the Carbon Dioxide Information and Analysis Center (CDIAC) ^7^ which extend through 2008. The emissions in 2009 are extrapolated using energy consumption statistics from the BP Statistical Review of World Energy 2011. The spatial patterns of fossil fuel emissions within each country are from the EDGAR v4.0 inventories ^8^. The biomass burning emission is from the fPAR-driven Global Emissions Fire Database (GFED 3.1) ^9^.

Prior fluxes of terrestrial ecosystems are simulated using the BEPS model ^10, 11^, and the ones of global ocean are calculated using the air-sea CO_2_ partial pressure difference of ocean interior inversion calculations ^12^. The 1σ uncertainties for the prior fluxes over the global land and ocean surfaces are assumed to be 2.0 and 0.67 PgC yr^-1^, respectively, which are the same as those used in Deng and Chen ^13^. The uncertainty on land is spatially distributed based on the annual NPP distribution simulated by BEPS, while it is distributed over the ocean surface according to the area of each ocean region. The monthly uncertainties for the terrestrial regions are assigned according to a profile combined using the variations of monthly NPP and soil respiration (RESP) ^14^, while the ones for oceanic regions are assumed to be even. We do not consider the relationship among different regions. Hence, a diagonal matrix for error variances is used. That is because the global land is separated into a series of regions mainly according to land cover types, and we assume that the relationship of the fluxes of different land cover types could be negligible.

***2) CarbonTracker-China system***

CarbonTracker China (CTC) ^15^ is a China-focused version of CarbonTracer (CT) ^5^. CT is global carbon assimilation system, which was designed to estimate global and regional net surface carbon fluxes by minimizing the Euclidean distance between the simulated and the observed CO_2_ concentrations using an ensemble fixed-lag Kalman smoother ^16^. In CT, the surface fluxes can be further divided into 4 categories as follows:

(4)

where *F_bio_*, *F_oce_*, *F_ff_* and *F_fire_* represent the prior fluxes of land biosphere and ocean, and carbon emissions from fossil fuel combustion and biomass burning, respectively. The ocean fluxes used in CT (*F_ocn_*) are the same as those used in BI, while the other three fluxes are different from those of BI. In CT, *F_bio_* is calculated using the Carnegie-Ames-Stanford approach (CASA)-GFED2 biogeochemical modeling system ^17^. *F_fire_* is obtained from the Global Emissions Fire Database version 2 (GFED2) ^18^ which extend through 2008, and the emission in 2009 is calculated using the climatological average. *F_ff_* is obtained from the “Miller” emissions dataset as well, but from a previous version, which is the same as those used in CarbonTracker CT2010 ^19^.andare scaling factors of land and ocean regions. There are 126 terrestrial regions (*N_eco_*) and 30 oceanic regions (*N_oce_*). For the global terrestrial regions, they were firstly derived based on the 11 TransCom large regions ^2^, and then for each large region, it was further separated according to land cover type.

The atmospheric transport in CTC is also simulated using TM5, which was run at a global horizontal resolution of 6° × 4° with a nested grid of 3° × 2° over Asia and a further nested grid of 1° × 1°over China.

CT has been successfully applied to estimating global and regional carbon fluxes, especially in North America, Europe, and East Asia ^5, 15, 20^.

**S1.2 Observations and model-data mismatch errors**

***1) Global published CO_2_ dataset***

Global published CO_2_ datasets include the CO_2_ products of GLOBALVIEW (GV)-CO_2_, Observation Package data products (http://www.esrl.noaa.gov/gmd/ccgg/obspack/) and the World Data Centre for Greenhouse Gases (WDCGG, http://ds.data.jma.go.jp/gmd/wdcgg/). In the BI system, 130 sites of monthly CO_2_ concentrations from GLOBALVIEW-CO_2_ 2010 ^21^ are used, and in the CTC system, 95 time series from the ObsPack dataset (v1.02) and 4 stations from the WDCGG are included. It should be noted that the observation sites included in GV and ObsPack are basically the same, but GV is a data product derived from atmospheric measurements using data extension and integration techniques ^22^, hence it contains no actual data, while the datasets of ObsPack and WDCGG are both actual data. Table S1 lists the observation sites in and around China used in both BI and CTC system. More details about the global CO_2_ dataset used the BI and CTC systems are found in Jiang et al. ^23^ and Zhang et al. ^15^, respectively.

1. *CO_2_ measurements from China* ***Meteorological Administration***

Surface weekly flask CO_2_ measurements from Jul 2006 to Dec 2009 at 3 sites operated by Chinese Academy of Meteorological Sciences, China Meteorological Administration (CAMS/CMA) ^24, 25^ are used in this study. The 3 CAMS/CMA sites are located in Northeast China (LFS), North China (SDZ), and East China (LAN), respectively. They are all regional background stations. The measurements in these stations are sampled and analyzed using the recommended methods of WMO/GAW, and the accuracy is comparable with that of NOAA/ESRL. It should be noted that because the BI system uses smoothed data, the flask data are averaged and smoothed using the same method as that used for processing GV data ^22^ in order to be consistent with GV.

1. *CONTRAIL Aircraft CO_2_ measurements*

Aircraft CO_2_ measurements from Nov 2005 to Dec 2009 over Eurasian by the Comprehensive Observation Network for Trace gases by AirLiner (CONTRAIL) project ^26, 27^ are also used in this study. CONTRAIL CO_2_ data are measured using continuous measurement equipment mounted on passenger aircrafts with the NIES 09 CO_2_ scale, which are lower than the WMO-X2007 CO_2_ scale by 0.07 ppm at around 360 ppm and consistent in the range between 380 and 400 ppm ^28^. Hence, the accuracy of CONTRAIL data compares well with the surface data. The continuous measurements are firstly divided into a series of discrete observation sites before being put into the inversion systems ^14, 29^. In the BI system, the measurements are divided into 87 sites, including 19 level flight sites (~10 km) and 68 vertical sites. Following Niwa et al. ^30^, each vertical measurement profile is divided into 5 layers: 0 ~ 1000m, 1000~2000m, 2000 ~4000m, 4000~6000m, and 6000~8000m. Flights with altitudes higher than 8000 mare considered as level flights. The same as CAMS/CMA measurements, before being used in the BI system, the data of each site are smoothed, and then averaged to monthly mean values. In the CTC system, the measurements are divided into 4 layers: 575–625,465–525, 375–425, 225–275 hPa. The 4^th^ layer (225-275 hpa) is from leveling cruise. More details about the treatments of the CONTRAIL data are found in Jiang et al. ^14^ and Zhang et al. ^29^.

1. *Model-data mismatch errors*

The estimation of the model-data mismatch is very difficult ^31^, since the errors come from both observations (instrument errors) and simulations. Various methods ^1, 32, 33^ have been used to determine the model-data mismatch.

In the BI system, the model-data mismatch error in ppm is defined using the following function, which is similar to those used by Peters et al. ^16^ and Deng and Chen ^13^.

(5)

where *GVsd* reflects the observation error, for the GV data, it is the standard deviation of the residual distribution in the average monthly variability (var) file of GLOBALVIEW-CO_2_ 2010, and for the CMAS/CMA and CONTRAIL data, it is the standard deviation of the residual after smoothing the daily CO_2_ concentrations using the method of Masarie and Tans ^22^. The constant portion σ*_const_* reflects the simulation error, which varies with station type because transport models generally have different performances at different observation stations. Except for some difficult stations, the observation sites are divided into 5 categories. The categories (respective value in ppm) are: Antarctic sites/oceanic flask and continuous sites (0.30), ship and tower sites (1.0), mountain sites (1.5), aircraft samples (0.5), and land flask/continuous sites (0.75). The value of 3.5~5 is used for the difficult sites (e.g., abp_01D0, bkt_01D0). The CAMS/CMA sites are treated as difficult sites in this study, because all the three sites are regional background stations: SDZ is near Beijing City, LAN is near Yangtze River Delta (about 50 km from Hangzhou), and LFS is near Harbin city (120 km). For the CONTRAIL data, we use a constant of 0.75.

In the CTC system, the model-data mismatch errors are classified into six categories: (1) marine boundary layer (0.75 ppm), (2) land stations (2.50 ppm), (3) mixed stations (1.50 ppm), (4) aircraft measurements (2.00 ppm), (5) tower stations (3.00 ppm), and difficult sites (7.5 ppm). Similarly, the CAMS/CMA sites are treated as difficult sites, with model-data mismatch error of 7.5 ppm. It should be noted that we discard some observations in categories 2–6 in our assimilation when the residuals (observed-simulated) exceed 3 times of the model-data mismatch error.

S1.3 Experiments

In order to quantify the impact of the additional CO_2_ data on the inversion results in China, 6 experiments are conducted with BI and CTC systems, as follows:

BI_Case_1: using the BI system, constrained using the 130 GLOBALVIEW sites;

BI_Case_2: the same as BI_Case_1 but with additional observations from the 3 CAMS/CMA sites;

BI_Case_3: the same as BI_Case_2 but with additional constraint from the CONTRAIL CO_2_ data;

CTC_Case_1: using the CTC system, constrained using the 99 observation sites from ObsPack and WDCGG datasets;

CTC_Case_2: the same as CTC_Case_1 but with additional observations from the three CAMS/CMA sites;

CTC_Case_3: the same as CTC_Case_2 but with additional constraint from the CONTRAIL CO_2_ data;

Both the BI and CTC systems are run from 2000 to 2009. Since both the CAMS/CMA and CONTRAIL data are from 2006 to 2009, the inverted results from 2006 to 2009 from both systems are used for analysis.

*S1.4 Inversion Results*

The inverted global net fluxes during 2006 – 2009 from BI_Case_1 and CTC_Case_1 are 4.15 and 4.00 PgC yr^-1^, respectively, which are close to other inversion estimates of 4.0 PgC yr^-1^ by Rödenbeck ^34^, 3.88 PgC yr^-1^ by CT2011_oi and 4.20 PgC yr^-1^ by Chevallier et al. ^35^ during the same period, but slightly higher than the atmospheric CO_2_ growth rate of 3.86 PgC yr^-1^ reported in the Global Carbon Budget 2013 ^36^.The global terrestrial ecosystem carbon fluxes from BI_Case_1 and CTC_Case_1 are -2.71 and -3.32 PgC yr^-1^, respectively. The result of BI_Case_1 is lower than the values of -3.13 PgC yr^-1^ by Le Quéré et al. ^36^, while the result of CTC_Case_1 is slightly higher than the Le Quéré’s result. Both values of BI_Case_1 and CTC_Case_1 are adjusted with the same fire and fossil fuel emissions as Le Quéré et al. ^36^.

The inverted carbon sinks (excluding CO_2_ emissions from fossil fuel) in China during 2006 – 2009 from BI_Case_1 and CTC_Case_1 are -0.29 ± 0.20 and -0.21 ± 0.36 PgC yr^-1^, respectively. Since the BI and CTC use different fossil fuel emissions, in order to do comparison, these sinks have been adjusted with the national CO_2_ emissions from fossil fuel burning, cement manufacture, and gas flaring of 1.90 PgC yr^-1^during 2006 – 2009 reported by the Carbon Dioxide Information Analysis Center ^37^. These values are close to pervious inversion estimates of -0.16 PgC yr^-1^ by CarbonTracker-EU ^20^ and -0.34 PgC yr^-1^ by CarbonTracker-US 2011_oi ^5^ for the same period, -0.35 ± 0.33 PgC yr^-1^ by Piao et al. ^38^ for 1995 –2005, and -0.28 PgC yr^-1^ by Rayner et al. ^39^ for 1979–1999. Piao et al. ^38^ used the Bayesian synthesis inverse method as well, but they did inversion for monthly fluxes in a 3.75^o^×2.5^o^ global grid system (i.e., 96 × 72 grids for globe). The result of Rayner et al. ^39^ is generated by a carbon cycle data assimilation system, which was also inverted using the Bayesian approach. In addition, it should be noted that the CT systems uses global raw data, while the BI system of this study and those of Piao et al. ^38^ and Rayner et al. ^39^ use monthly mean data (i.e. GLOBALVIEW-CO_2_). Overall, these results are inverted using very different systems, but the results from these systems are very close to each other. These means that the inverted carbon sinks in China are mostly in the range of -0.21 to -0.35 PgC yr^-1^ if only constrained by the global published CO_2_ datasets.

When CMA observations are added to the BI and CTC systems, the inverted carbon sink in China increases to 0.43± 0.19 and 0.29 ± 0.36 PgC yr^-1^, respectively, and when both CMA and CONTRAIL data are added, the inverted sinks further increases to 0.51 ± 0.18 and 0.39 ± 0.33 PgC yr^-1^, respectively. The increases of land sink mainly occur in eastern part of China (Figure S3). After constraint with additional data, the spatial patterns of the inverted land sinks from both systems are similar with each other, and are basically consistent with the eddy-covariance observations by ChinaFlux (Figure S4) reported by Yu et al. ^40^, who found that there is high CO_2_ uptake in the East Asian monsoon region, including eastern China. Large uncertainty may exist in south and southwest China, one of the largest forest areas in China, since there are significant differences in the inverted fluxes from the two systems, especially for the fluxes in Guizhou and Chongqing provinces; and the inverted fluxes from both systems are obviously lower than the fluxes observations in Yunnan and Guangdong provinces (Figure S4). That is because there is no surface CO_2_ observation in south and southwest China, and the carbon flux inversion over these regions cannot be reliably constrained using CO_2_ measurements at other stations because very few air masses from these regions move to existing observation stations (Figure S5). In addition, the inverted carbon sinks over the western grass areas may be overestimated as well, that may should be attributed to the coarse inversion resolution, since there is a considerable proportion of forest in this region as well.

***S2. Simulating the soil carbon fluxes of forest land***

The Integrated Terrestrial Ecosystem C-budget (InTEC) model is a regional C-budget model, which combines the CENTURY model for soil C and nutrient dynamics ^41, 42^ and Farquahar’s leaf biochemical model for canopy-level annual photosynthesis ^43, 10^ implemented using a temporal and spatial scaling scheme ^44, 45^. In the InTEC model, there are 4 biomass C pools (wood, leaf, coarse root, and fine root),9 soil C pools (woody litter, soil metabolic detritus, soil structural detritus, surface structural detritus, surface metabolic detritus, soil microbe, surface microbe, slow and passive), and 3 forest product C pools (fuelwood, paper products and long-term storage). InTEC simulates the C pool changes caused by C fluxes among these pools and between the pools and the atmosphere. The major inputs for the model include: historical CO_2_ concentrations; maps of NPP, LAI, stand age, land cover type, nitrogen deposition, and evapo-transpiration in the reference year; historical climate datasets such as temperature in the growing season, the length of the growing season, annual mean temperature, and annual precipitation; and soil texture. A detailed description about this model can be found in Chen et al. ^45^, ^46^.

In this study, the InTEC model is run from 1901 to 2012. The simulation region covers the whole China, with a horizontal resolution of 1 km × 1 km. The data used to drive the model are as follows: 1) LAI data in 2005 (8 days interval, 500 m resolution), which were derived from the Moderate Resolution Imaging Spectroradiometer (MODIS) reflectance and land cover products ^47^; 2) forest cover data (1 km resolution) of 2005, which were obtained from the Institute of Remote Sensing and Digital Earth of Chinese Academy of Sciences; 3) stand data (1 km resolution) generated using the remotely sensed forest height and forest type data in 2005, as well as relationships between age and height retrieved from field observations ^48^; 4) climate data including mean temperature, precipitation, and water vapor pressure (0.5° resolution) during 1901 – 2012, which were generated by the University of East Anglia Climatic Research Unit, downloaded from the website of http://www.cru.uea.ac.uk/; 5) nitrogen deposition data (0.1° resolution) during 1901-2010, which was obtained from the Institute of Environment and Sustainable Development in Agriculture, Chinese Academy of Agricultural Sciences; 6) soil data (0.00833° resolution) downloaded from http://globalchange.bnu.edu.cn/research/, which were combined based on the second national soil survey data ^49^; 7) NPP data in the reference year of 2005 simulated using the BEPS model ^47^; 8) historical mean CO_2_ concentrations in China during 1901 - 2012, and for 1901 - 1998, the data were from the Carbon Cycle Model Linkage Project, while for 1999-2012, the data from the Mauna Loa site were used directly.

The simulated soil carbon accumulation rate of forestland from 2006 to 2009 is 0.068 ± 0.034 PgC yr^-1^. This 50% uncertainty is quoted according to previous modelling studies ^50^ and a model validation ^51^. Shao et al. ^51^ simulated soil organic carbon (SOC) density using InTEC model at two sites (Liping and Changbaishan) and evaluated the simulated results using the observed ones at these two sites. The two sites are located in China's northern temperate zone and southern subtropical zone, respectively. Their results showed that the simulated SOC density were highly correlated and in broad agreement with observations in Liping and in Changbaishan, with the correlation coefficients (r^2^) of 0.63 (N=16) and 0.76 (N=14) between the simulated and measured data in Liping and Changbaishan, respectively. This successful test of the InTEC model without modifying the decomposition coefficients of the various carbon pools in these two contrasting climate conditions gave us a strong confidence for the performance of the InTEC model to all of China's forests. Wang et al. ^50^ modeled the carbon sinks and sources in China’s forests during 1901-2001 using InTEC as well, and they pointed out that over the entire 1901–2001 period, the uncertainty of NEP simulation reached 100%, but during 1988-2001, this uncertainty decreased to about 25 – 40%. Therefore, we give a conservative estimation of uncertainty about 50% in this study.

***S3. Reduced carbon compounds emissions in China***

The reduced carbon compou nds (RCC) include CH_4_, CO, and NMVOCs, with sources of fossil fuel, biomass burning and other biogenic. Based on the Asian emission inventory for 2006 for the NASA INTEX-B Mission ^52^, and the Multi-resolution Emission Inventory for China (MEIC) ^53^ (available at http://meicmodel.org) for 2008 and 2010, the fossil fuel emissions of CO and NMVOCs during 2006 - 2009 in China are calculated to be 72.4 and 18.7 TgC yr^-1^, respectively. Based on Global Fire Emission Data (GFED) V3.1 ^9^, the biomass burning emissions of CO and NMVOCs in China are calculated to be 1.0 and 0.1 TgC yr^-1^, respectively. The biogenic NMVOCs emissions were estimated to be in the range from 12.4 to 28.4 TgC yr^-1^ in previous studies ^54, 55, 56, 57^. In this study, we use the estimate of 21 TgC yr^-1^ reported by Klinger et al. ^55^. CH_4_ could be emitted from fossil fuels, biomass burning, agricultural activities, waste management, wetlands and some other sources. Based on the top-down estimate reported by Kirschke et al. ^58^, the emissions from fossil fuels, biomass burning, and other biogenic sources are 11.3, 0.8, and 27 TgC yr^-1^, respectively, with total emission of 39 TgC yr^-1^. Generally, the uncertainty in the biogenic RCC emissions may be greater than 50% in China ^59^. In this study, we assume that there is an uncertainty of 50% for the biogenic RCC emissions. For the uncertainty in fossil fuel RCC emission, we assume it has the same uncertainty with fossil fuel CO_2_ emission, which is about 7% ^60^; and for the uncertainty in biomass burning, we do not consider it, since this emission is rather lower compared with the ones from other biogenic sources and fossil fuels. Therefore, the total RCC emission in China is 151.2 ± 31.0 TgC yr^-1^, in which 102.3 ± 7.0 TgC yr^-1^ (67.7%) are from fossil fuels, and 49.9 ± 24 TgC yr^-1^ (32.3%) are of biogenic origin.

Based on the simulation using a global 3-D chemistry transport model by Ciais et al. ^61^, 14% of the RCC emissions are oxidized to CO_2_ in the boundary layer of Europe, 12% of the RCC are deposited to land surface, and the other (74%) are transported to global atmosphere. In this study, we directly use these fractions to estimate the transformation of RCC in China. As shown in Table S2, we estimate the CO_2_ production from RCC from fossil fuel and biosphere (include biomass burning and biogenic emission) in the boundary layer are 14.3 ± 1.0 and 7.0 ± 3.4 TgC yr^-1^, respectively, and the RCC from fossil fuels that transfer to the global atmosphere is 76 ± 5.3 TgC yr^-1^. The CO_2_ production in the boundary layer has been included in the inversions, but we do not consider these contributions from biomass burning and biogenic emissions, which may lead to an underestimation of 7.0 ± 3.4 TgC yr^-1^for the land sink obtained by atmospheric inversions. However, for the RCC from fossil fuel, since the fossil fuel emission inventories used in the inversions have treated the RCC as direct emission of CO_2_, which may lead to an overestimation of 87.7 ± 6.1TgC yr^-1^for the land sink. Totally, these two items lead to an overestimation of 80.7 ± 7 TgC yr^-1^ for the inverted land sink. The RCC emitted from biosphere originates from the carbohydrate fixed by plants, which would reduce the carbon pools of vegetation and soil, while the RCC deposited to the land surface would increase the carbon pools of soil. As a result, the RCC emission and deposition would lead to an underestimation of 32.1 ± 20 TgC yr^-1^ for the carbon accumulation rate of vegetation and soil estimated using bottom-up methods.

***S4. Export of carbon by Chinese rivers to the ocean***

***Net transport to the ocean***

In China, the main exorheic rivers include Yangtze River, Yellow River, Pearl River, Huai River, Hai River, Liao River, Songhua River, Qiantang River, and Min River. Yangtze River, Yellow River and Pearl River are the top 3 rivers in China in terms of their discharge volumes. In recent years, a series of studies have been conducted to investigate the riverine carbon transport in the estuaries of these rivers ^62, 63, 64, 65^. For example, Ran et al. ^63^ reported that during 2008 - 2012, 0.06TgC yr^-1^ of DOC, 0.41TgC yr^-1^ of POC and 1.09TgC yr^-1^ of DIC were transported to ocean by Yellow River. The total DOC, POC, and DIC export by the top 3 rivers are measured to be 2.09, 5.11 and 23.74 TgC yr^-1^, respectively (Table S3). In addition, Xia and Zhang ^65^ studied the carbon fluxes of Hai River and Liao River basins in summer 2005. They estimated that the DOC, POC and DIC fluxes from these two rivers discharging into the Bohai Sea in the summer of 2005 were 0.06, 0.086 and 0.383 TgC, respectively. Considering the fact that the precipitation in North China mainly occur in summer, we assume that the annual carbon transport by these two rivers are 2 times of those in summer. Therefore, the annual transport of DOC, POC, and DIC by Hai River and Liao River were 0.12, 0.17 and 0.77 TgC yr^-1^, respectively. For the other 4 rivers in China, until now, there is no observation. We use several simple methods to estimate the transport. For DOC, we assume that the DOC concentrations in the other 4 rivers are similar with the ones in Yellow and Yangtze River. Ran et al. ^63^ measured that the mean DOC concentration in the estuary of Yellow River was 3.3 mg L^-1^, which was comparable with the one of Yangtze River ^66^, but slightly lower than the global mean of 4.9 mg L^-1 67^.Therefore, we apply this concentration of 3.3 mg L^-1^ to the other rivers, and calculate that about 0.48 TgC yr^-1^ of DOC are transported to the ocean by the other 4 rivers. For DIC, we simply calculate using the mean DIC/DOC ratio observed in Yellow River, Yangtze River, Pearl River, Hai River and Liao River. The DIC/DOC ratios in the 4 rivers are in the range of 5.6 ~ 18.1, with a mean of 11. Hence, we estimate that the amount of DIC exported to ocean by the other 4 rivers is about 5.26 TgC yr^-1^. For POC, generally, the concentration of POC is related to the concentration of total suspended solids (TSS), and Ludwig et al. ^68^ found an empirical formula to express the relationship between the POC/TSS ratio and TSS concentration, as follows:

$POC\%=-0.16\times(\log C_{TSS})^{3}+2.83\times(\log C_{TSS})^{2}-13.6\times\log C_{TSS}+20.3$ (6)

where *POC%* is the percentage of POC in TSS, *C_TSS_* is the concentration of TSS. This formula is applicable to rivers with C*_TSS_* less than 2250 mg L^-1^. Using this empirical formula, Zhu et al. ^69^ calculated the riverine fluxes of POC in China during 1965-2005. Following Zhu et al. ^69^, we use this formula to estimate the exports of POC by Huai River, Songhua River, Qiangtang River and Min River during 2006 -2009 in this study. The data of *C_TSS_* is from China's river sediment bulletin ^70, 71, 72, 73^. We estimate the amount of POC exported to ocean by the 4 rivers is about 0.205 TgC yr^-1^. In total, the DOC, POC and DIC transported to the ocean by all major Chinese rivers are 2.69, 5.48 and 29.76 TgC yr^-1^, respectively.

***CO_2_ outgassing from inland water***

The CO_2_ outgassing from inland waters in China is calculated based on limited observations of CO_2_ outgassing rates (CORs) in Chinese rivers. The CORs in Pearl River, Yangtze River and Yellow River have been studied in the past decade. The CORs observed in Xijiang River (the main branch of Pearl River) and Yangtze River were in the range of 830-1560 gC m^-2^yr^-1 74^, and in the range of 186-1585 gC m^-2^ yr^-1^ ^62, 75 , 76 , 77^, with mean values of 1195 ± 365 and 886 ± 669 gC m^-2^yr^-1^, respectively, and the one observed in Yellow River was in the range of 386.4-1375.2 gCm^-2^yr^-1^, with mean of 881 ± 494 gCm^-2^yr^-1^ ^63^. Therefore, we use an average rate of 987 ± 509 gCm^-2^yr^-1^ for Chinese rivers, which is much lower than the average flux of 2370 gC m^-2^yr^-1^ in the US ^78^. Based on the data of National Bureau of Statistics in China, the water surface area of the rivers and streams is 52780 km^2 79^, as the CO_2_ outgassing in Chinese rivers and streams is calculated to be 52.1 ± 27 TgC yr^-1^. Moreover, the lakes and reservoirs in China also act as a source of CO_2_ ^80^, the COR of natural lakes is 70 gC m^-2^yr^-1 77^, and the one of reservoirs in China is 175.2 ± 118 gC m^-2^yr^-1 81^, which is close to the global temperate reservoirs mean of 139 gC m^-2^ yr^-1 82^. The water surface area of lakes and reservoirs is 75240 and 23020 km^2 79^, respectively. So, we calculate the CO_2_ outgassing by Chinese lakes and reservoirs are about 5.27 and 4.03 ± 2.72 TgC yr^-1^, respectively. In total, the amount of CO_2_ outgassing in inland waters in China is calculated to be 62 ± 30 TgC yr^-1^.

***Carbon burial in lakes and reservoirs***

Gui et al. ^83^ studied the organic carbon burial in six lakes in the middle and lower reaches of the Yangtze River Basin, and showed that the mean organic carbon burial rates during 2000swerein the range of 20.9 to 312.2 gC m^-2^yr^-1^, except for Honghu Lake (312.2 gC m^-2^yr^-1^), the carbon burial rates of the other five lakes are all around 30 gC m^-2^ yr^-1^, with mean of 31 gC m^-2^yr^-1^. Dong et al. ^84^ studied the carbon burial of floodplain lakes in the middle and lower reaches of the Yangtze River Basin during 2003 - 2004, and their result showed that the carbon burial rates ranged from ~5 to 373 g C m^-2^ yr^-1^, and generally, larger lakes have smaller burial rates. Based on the data reported in Dong et al. ^84^, we calculate the area weighted mean of burial rate of all lakes with area > 1 km^2^ is about 26 gC m^-2^yr^-1^. The mean rate of these two studies is 28.5 gC m^-1^ yr^-1^, which is about two times of the global mean rate of 14 gC m^-2^yr^-1^ ^83^. Simply using this rate to calculate the carbon burial in all Chinese lakes, we estimate the value is about 2 TgC yr^-1^. For the reservoirs in China, due to lack of observations, we assume that the carbon burial rate in Chinese reservoirs is also about two times of the global mean rate (400 gC m^-2^yr^-1^) ^85^, and estimate that the carbon burial in Chinese reservoirs is about 18 TgC yr^-1^. Therefore, the total carbon burial in lakes and reservoirs in China is estimated to be 20 TgC yr^-1^.

***Carbon balance in inland waters in China***

Based on above estimates, the carbon balance of inland waters in China could be illustrated (Figure S7). Since the uncertainty of CO_2_ outgassing from inland waters is about 50%, though the ones of carbon burial and exported to ocean are not estimated, we assume that they also have an uncertainty of 50%. Overall, inland waters in China annually receive 120 ± 60 TgC yr^-1^ of carbon, in which 105 ± 50TgC yr^-1^is from terrestrial landscape, and 15 ± 7.5 TgC yr^-1^ is from the rock weathering (half of the exported DIC ^61^). During the transport processes, about 20 ± 10 TgC yr^-1^ of carbon is buried in aquatic sediments, and 62 ± 30 TgC yr^-1^ is returned to the atmosphere, and the remaining of 38 ± 19 TgC yr^-1^ is delivered to ocean.

***S5. Trade of food***

Using the same method as Piao et al. ^38^, we use the import and export data of food products from Food and Agriculture Organization of the United Nations (FAO) statistical databases ^86^, and recalculate the net carbon import by international trade of food. The food products include cereals, roots, sugar, soyabeas and pulses, oil crops, vegetables, fruits, coffee and teas. We calculate that the net carbon import of food products during 2006 – 2009 was 0.019 PgC yr^-1^(Table S6). This estimate is much higher than that of Piao et al. (0.004 PgC yr^-1^).The increase of food carbon is mainly attributed to the significantly import increase of soybeans, because from 1995 to 2010, the net import of soybeans increased by 22.7 times.

**S6. Trade of wood and carbon pool changes of wood products**

Using the production, import and export data of wood products from Food and Agriculture Organization of the United Nations (FAO) statistical databases ^86^, we calculate the net carbon import and CO_2_ emissions by international trade of wood, and the carbon pool changes of wood products due to local production. The wood products include sawnwood, wood-based panels, paper and paperboard, recovered paper, other industry roundwood, and wood fuel and charcoal. The data from 1961 to 2009 were used in this study.

Except for the paper products and wood charcoal, the other products in the FAO database are reported in volume units. We use a series of conversion factors adopted from Winjum et al. ^87^ to convert the products into dry mass. They are 0.42 for conifer sawnwood, 0.53 for non-confier sawnwood, 0.52 for wood-based panels, 0.6 for other industry roundwood, and 0.45 for wood fuel. Then, the dry masses of wood products are converted to C contents using a common factor of 0.5. The CO_2_ release and the carbon accumulation due to international trade and local production in China are calculated according to the method of Winjum et al. ^87^. Every year, the wood products of wood fuel and charcoal are assumed to be totally consumed and oxidized to CO_2_, while the products of sawnwood, wood-based panels, paper and paperboard, and other industry roundwood, are partially oxidized to CO_2_ in the same year and the others go into uses or long-term storage for 5 years or more. The proportions that go into long-term uses are: sawnwood, 0.8; wood-based panels, 0.9; paper and paperboard, 0.6; other industry roundwood, 0.7. The wood in long-term use is assumed to be slowly oxidized at a constant rate over time. The oxidation fractions for sawnwood, wood-based panels, paper and paperboard, other industry roundwood are 0.01, 0.02, 0.01 and 0.04 yr^-1^, respectively. It should be noted that a part of the paper and paperboard that go into long-term use are recovered for paper production again. In order to avoid double counting, when we calculate the production of paper and paperboard, the recovered paper is subtracted.

Figure S7 shows the time series of local production and international trade of wood and their uses from 1961-2009. The wood production in China increases from 1961 to 1976, and remains stable from 1976 to 1990, and then decreases from 1990 to 2001, after 2001, it is significantly increased again. For the wood products produced in China, although the easy oxidation part is dominant, its proportion is reduced year by year from 1967 to 2009, while the proportion of long term use continue to increase after 1976, especially after 2001. Here, the easy oxidation part includes wood fuel and charcoal, and a small proportion of sawnwood, wood-based panels, paper and paperboard, and other industry roundwood. After 1976, China has been to a net timber importer, the amount of net import significantly increases after 1980. In the imported timber, the long term use part is dominant. After 2000, the import still keep increasing, although it is reduced in 2005, after that, it is rapidly increasing again. The rapidly increasing of local production and import of wood products after 2000 are related to the rapidly urbanization in China during the same period. Accordingly, the wood product carbon pool increases as well (Figure S8). During 2000s, the mean increasing rate of carbon pool from local wood production is 4.8 TgC yr^-1^. Due to the increase of wood import, the carbon emission from wood import is also increasing, with mean emission rate of 6 TgC yr^-1^ during 2006 -2009. Therefore, the accumulation rate of wood products in China is about 0.005 PgC yr^-1^, which is slightly lower than the estimate of 0.007 PgC yr^-1^ by Pan et al. ^88^ and the estimate of 0.01 PgC yr^-1^ by Piao et al. for 1996-2005.

**S7. Adjusting from inverted CO_2_ sink to land sink**

*Land sink* _top-down_ = **inverted CO_2_ sink** – fossil fuel RCC transferred to global atmosphere – fossil fuel RCC deposited to land surface - biogenic RCC transferred to global atmosphere + net import (7)

The above equation was derived as follows:

First, in order to simplify the above formula, we define a series of variables, as follows:

*NEP*: a net contribution of photosynthesis and respiration of land ecosystems.

*FFC*: CO_2_ emissions due to fossil fuel combustion

*FFRCC*: RCC emissions due to fossil fuel combustion

*FFRCC*_o_: FFRCC oxidized in China’s boundary layer

*FFRCC*_t_: FFRCC transferred to global atmosphere

*FFRCC*_d_: FFRCC deposited to land surface

*BBC*: CO_2_ emissions due to biomass burning

*BRCC*: biogenic RCC emissions due to biomass burning and biogenic VOC emissions

*BRCC*_o_: BRCC oxidized in China’s boundary layer

*BRCC*_t_: BRCC transferred to global atmosphere

*BRCC*_d_: BRCC deposited to land surface

*NIC*: net import due to international trade

*COW*: CO_2_ outgassing from inland water surface

*NCF*: net carbon flux

*LCS*: land CO_2_ sink derived using top-down method.

*ICS*: inverted CO_2_ sink

All above variables are positive.

As, equation (7) could be shortened to

***LCS* = *ICS – FFRCC*_t_ *– FFRCC*_d_ *- BRCC*_t_ *+ NIC***

Then, from the atmospheric view, the net CO_2_ flux of one specific region includes fossil fuel CO_2_ emissions, biomass burning CO_2_ emissions, CO_2_ outgassing from the inland water surface, CO_2_ emissions from net imported products, CO_2_ conversions from RCC, CO_2_ release by respiration and CO_2_ uptake by photosynthesis. Therefore, we can get a formula as follow,

*NCF* = *FFC* + *FFRCC*_o_ + *BBC* + *BRCC*_o_ + *COW* + *NIC* – *NEP*

🡺

*NEP* = *FFC* + *FFRCC*_o_ + *BBC* + *BRCC*_o_ + *COW* + *NIC* – *NCF* (8)

Since,

*BRCC*_o_ = *BRCC* –*BRCC*_t_ - *BRCC*_d_

*FFRCC*_o_ = *FFRCC* –*FFRCC*_t_ - *FFRCC*_d_

So, equation (8) could be rewritten as:

*NEP* = *FFC*+ (*FFRCC*-*FFRCC*_t_-*FFRCC*_d_) + BBC + (*BRCC*-*BRCC*_t_ - *BRCC*_d_) + *COW* + *NIC* - *NCF*

🡺

*NEP* = (*FFC* + *FFRCC* -*NCF* ) + *BBC* + *BRCC* - *BRCC*_t_ - *BRCC*_d_ - *FFRCC*_t_ - *FFRCC*_d_ + *WOC* + *NIC* (9)

In the inversion systems used in this study, the carbon sink was estimated by excluding CO_2_ emissions from fossil fuels while sources from biomass burning are included. In addition, the fossil fuel carbon emission inventories used in the inversion systems are based on CO_2_ emission factors that include direct emissions of CO_2_ from fossil fuels and emissions of RCC. Therefore, the inverted CO_2_ sink (*ICS*) could be represented as follows:

*ICS* = *FFC* + *FFRCC* – *NCF* (10)

Combining equations (9) and (10), we can get

*NEP* = ***ICS*** + *BBC*+ *BRCC* - *BRCC*_t_ - *BRCC*_d_ - *FFRCC*_t_ - *FFRCC*_d_ + *COW* + *NIC* (11)

Next, from the terrestrial view, the land sink could be defined as net CO_2_ uptake by photosynthesis and respiration of land ecosystems plus RCC deposited to the land surface minus biogenic carbon emissions (including biomass burning emission and biogenic emission) and CO_2_ outgassing from the inland water surface, which was firstly fixed by NEP, then transported to inland waters, and finally emitted from the water surface. i.e.,

*LCS* = *NEP* + *BRCC*_d_ – *BBC* – *BRCC* – *COW* (12)

Finally, combining equations (11) and (12), we can get

***LCS* = *ICS* - *BRCC*_t_ - *FFRCC*_t_ - *FFRCC*_d_ + *NIC*** (13)

**Table S1** Surface observation sites in and around China used in this study

| **Site Name** | **Location** | **Lat.** | **Lon.** | **Elev.** | **Lab.** | **Method** |
| --- | --- | --- | --- | --- | --- | --- |
| WLG | Mt. Waliguan, China | 36.29 | 100.9 | 3810 | ESRL | Flask |
| TAP | Tae-ahn Peninsula, South Korea | 36.73 | 126.12 | 20 | ESRL | Flask |
| GSN | Gosan, Cheju Island, South Korea | 33.28 | 126.15 | 72 | ESRL | Flask |
| KZD | SaryTaukum, Kazakhstan | 44.06 | 76.82 | 601 | ESRL | Flask |
| KZM^1)^ | Plateau Assy, Kazakhstan | 43.25 | 77.88 | 2519 | ESRL | Flask |
| UUM | UlaanUul, Mongolia | 44.45 | 111.10 | 914 | ESRL | Flask |
| HAT^2)^ | Hateruma Island, Japan | 24.05 | 123.80 | 47 | JMA | Continuous |
| YON | Yonagunijima, Japan | 24.47 | 123.02 | 30 | JMA | Continuous |
| RYO | RyoriBAPMon Station, Japan | 39.03 | 141.83 | 260 | JMA | Continuous |
| SDZ | Shangdianzi,China | 40.65 | 117.12 | 293 | CMA | Flask |
| LFS | Mt. Longfengshan, China | 44.73 | 127.60 | 330 | CMA | Flask |
| LAN | Lian, China | 30.42 | 119.73 | 132 | CMA | Flask |

^1)^only used in the CarbonTracker inversion system;

^2)^only used in the Bayesian inversion system

**Table S2** Reduced carbon compounds (RCC) emissions in China (TgC yr^-1^)

|  | Fossil fuel | Biomass burning | Biogenic emissions | Total |
| --- | --- | --- | --- | --- |
| CO | 72.4± 5.0 | 1.0 | 0.00 | 73.4± 3.6 |
| NMVOCs | 18.7± 1.3 | 0.1 | 21.0 ± 10.5 | 39.8± 11.4 |
| CH_4_ | 11.3± 0.8 | 0.8 | 27.0 ± 13.5 | 39.0± 14.1 |
| Total | 102.3± 7.1 | 1.9 | 48.0 ± 24 | 152.2±31.1 |
| Transform to CO_2_ in the boundary layer | 14.3± 1.0 | 0.3 | 6.7±3.4 | 21.3±4.4 |
| Deposit to the land surface | 12.0±0.8 | 0.2 | 5.6±2.8 | 17.8± 3.6 |
| Transfer to global atmosphere | 76.0±5.3 | 1.4 | 35.7±17.8 | 113.0±23.1 |

**Table S3** River carbon transport in China (TgC yr^-1^)

| River | Water | DOC | POC | DIC | Period | Ref. |
| --- | --- | --- | --- | --- | --- | --- |
| Yellow River | 25.2 | 0.06 | 0.41 | 1.09 | 2008-2012 | 63 |
| Yangtze River | 971 | 0.90 | 2.20 | 14.60 |  | 64 |
| Pearl River | 260 | 1.13 | 2.50 | 8.05 | 2012 | 89 |
| Hai River^a^ | 9.2 | 0.076 | 0.075 | 0.43 | 2005 | 65 |
| Liao River^a^ | 12.2 | 0.044 | 0.096 | 0.33 | 2005 | 65 |
| Huai River | 34.5 | 0.114 | 0.053 | 1.14 | 2006-2009 | This study |
| Songhua River | 42.3 | 0.140 | 0.070 | 1.40 | 2006-2009 | This study |
| Qiantang River | 17.5 | 0.058 | 0.037 | 0.58 | 2006-2009 | This study |
| Min River | 50.4 | 0.166 | 0.045 | 1.66 | 2006-2009 | This study |
| Total |  | 2.7 | 5.5 | 29.8 |  |  |

^a^the values reported in Xia and Zhang ^65^, in this study, we assume that the annual exports are two times of the summer ones.

**Table S4** Imports and exports of food and their carbon emissions in China during 2006 -2009

| Items | Imports (Gg yr^-1^) | Exports (Gg yr^-1^) | Net (Gg yr^-1^) | Biomass to dry matter | Dry matter to carbon | Carbon (TgC yr^-1^) |
| --- | --- | --- | --- | --- | --- | --- |
| Cereals^1)^ | 9651 | 6292 | 3359 | 0.85, 0.88 | 0.47~0.49 | 1.37 |
| Roots^2)^ | 18790 | 1048 | 17742 | 0.25, 0.38 | 0.44 | 2.96 |
| Sugar | 2046 | 927 | 1118 | 0.25 | 0.25 | 0.13 |
| Soyabeas and Pulses | 37873 | 1708 | 36165 | 0.85 | 0.48 | 14.76 |
| Oilcrops^3)^ | 2254 | 802 | 1452 | 0.50~0.95 | 0.45~0.60 | 0.45 |
| Vegetables | 1392 | 10585 | -9193 | 0.13 | 0.46 | -0.55 |
| Fruits | 3452 | 5836.25 | -2384 | 0.19 | 0.45 | -0.20 |
| Coffee and teas^4)^ | 165 | 350 | -184 | 0.13, 0.5 | 0.46, 0.6 | 0.01 |
| **Total** |  |  |  |  |  | **19.0** |

^1)^includes wheat, barley, maize, oats, rice, and other; the ratio of biomass to dry matter for rice is 0.88, and the others are 0.85; the ratios of dry matter to carbon for wheat and barley are 0.47, those for oats, rice, and other cereals are 0.48, and that for maize is 0.49.

^2)^includes potatoes, cassava, and other; the ratio of biomass to dry matter for cassava is 0.38, and the others are 0.25.

^3)^includes groundnuts, sunflower seed, rape and mustard seed, coconuts, sesame seed, and other, with the biomass to dry matter ratios of 0.95, 0.9, 0.85, 0.5, 0.85, and 0.5, respectively, and with the dry matter to carbon ratios of 0.6, 0.6, 0.48, 0.6, 0.54, and 0.45, respectively;

^4)^ includes cocoa beans and tea, with the ratios of biomass to dry matter and dry matter to carbon 0.5, 0.6 and 0.13, 0.46, respectively.

**Figure S1.** Locations of observation sites used in the inversions (red circle: sites only used in the CT system; black circle: sites only used in the BI system; green circle: sites used both in the CT and BI systems; blue solid circle: the 3 CAMS/CMA sites). The software GrADS v1.9b4 was used to create the map.


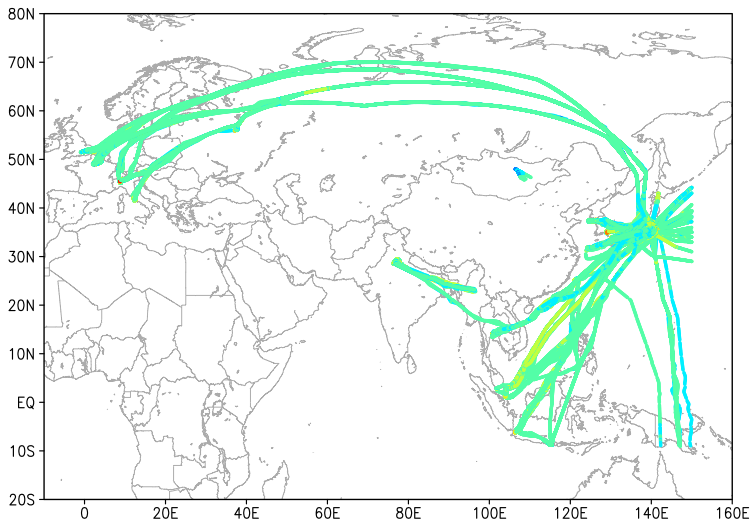


Figure S2. CONTRAIL aircraft CO_2_ measurements over Eurasia. The software GrADS v1.9b4 was used to create the map.

**Figure S3**. Distribution of mean annual carbon sources and sinks in China inverted using the Bayesian inversion system (BI) and the CarbonTracker-China (CTC) system (average from 2006 to 2009; gC m^-2^yr^-1^). The software GrADS v1.9b4 was used to create the map.

**Figure S4.** Evaluation for the spatial pattern of the inverted bio fluxes from (a) BI_Case_3 and (b) CTC_Case_3 with the ChinaFlux observations; shaded, inverted bio fluxes; cycle, ChinaFlux observations. The software GrADS v1.9b4 was used to create the map.

**Figure S5.** 5-day back trajectories of the 10 sites in and around China at 16:00 LST in 2006 (a, spring; b, summer; c, autumn; d, winter). Note that few tracjectories reach the observations sites from the southeast regions of China. The software GrADS v1.9b4 was used to create the map.

**Figure S6**. Carbon balance of inland waters in China (TgC yr^-1^)

**Figure S7.** Trend of local production and international trade of wood and their uses from 1961-2009

**Figure S8**. The carbon pool changes of wood products and total carbon emissions from wood products every year from 1961-2009

**Supporting References**

1. Rayner, P. J., Enting, I. G., Francey, R. J. & Langenfelds, R. Reconstructing the recent carbon cycle from atmospheric CO_2_, ^13^C and O_2_/N_2_ observations. *Tellus* **51B**, 213–232 (1999).
2. Gurney, K. R. *et al.* TransCom 3 CO_2_ inversion intercomparison: 1. Annual mean control results and sensitivity to transport and prior flux information. *Tellus* **55B**, 555–579 (2003).
3. Krol, M. *et al.* The two-way nested global chemistry-transport zoom model TM5: algorithm and applications. *Atmos Chem Phys* **5**, 417–432 (2005).
4. Meirink, J. F. *et al.* Four-dimensional variational data assimilation for inverse modelling of atmospheric methane emissions: Analysis of SCIAMACHY observations. *J Geophys Res* **113**, D17301 (2008).
5. Peters, W. *et al.* An atmospheric perspective on North American carbon dioxide exchange: CarbonTracker. *PNAS* **104**, 18925–18930 (2007).
6. CarbonTracker CT2011_oi, [http://carbontracker.noaa.gov](http://carbontracker.noaa.gov/) (2011).
7. Boden, T. A., Marland, G. & Andres, R. J. Global, regional, and national fossil-fuel CO_2_ emissions, Carbon Dioxide Information Analysis Center, Oak Ridge National Laboratory, US Department of Energy, Oak Ridge, TN, doi:10.3334/CDIAC/00001_V2011 (2011).
8. European Commission, Joint Research Centre (JRC)/Netherlands Environmental Assessment Agency (PBL). Emission Database for Global Atmospheric Research (EDGAR), release version 4.0 (2009).
9. Randerson, J. T. *et al.* Global Fire Emissions Database, Version 3 (GFEDv3.1). Data set. Available on-line [http://daac.ornl.gov/] from Oak Ridge National Laboratory Distributed Active Archive Center, Oak Ridge, Tennessee, USA. doi:10.3334/ORNLDAAC/1191 (2013).
10. Chen, J. M., Liu, J., Cihlar, J. & Goulden, M. L. Daily canopy photosynthesis model through temporal and spatial scaling for remote sensing applications. *Ecological Modelling* **124**, 99-119 (1999).
11. Ju, W. M. *et al.* Modelling multi-year coupled carbon and water fluxes in a boreal aspen forest. *Agricultural and Forest Meteorology* **140**, 136–151 (2006).
12. Jacobson, A. R. *et al.* A joint atmosphere-ocean inversion for surface fluxes ofcarbon dioxide: 1. Methods and global-scale fluxes. *Global Biogeochem Cycles* **21**, GB1019 (2007).
13. Deng, F. & Chen, J. M. Recent global CO_2_ flux inferred from atmospheric CO_2_ observations and its regional analyses. *Biogeosciences* **8**, 3263–3281 (2011).
14. Jiang, F. *et al.* Carbon balance of China constrained by CONTRAIL aircraft CO_2_ measurements. *Atmos Chem Phys* **14**, 10133-10144 (2014).
15. Zhang, H. F. *et al.* Net terrestrial CO_2_exchange over China during 2001–2010estimated with an ensemble data assimilation system for atmosphericCO_2_. *J Geophys Res Atmos* **119**, doi:10.1002/2013JD021297 (2014).
16. Peters, W. *et al.* An ensemble data assimilation system to estimate CO_2_ surface fluxes from atmospheric trace gas observations. *J Geophys Res* **110**, D24304 (2005).
17. Werf, G. *et al.* Interannual variability in global biomass burning emissions from 1997 to 2004. *Atmos Chem Phys* **6**, 3423–3441 (2006).
18. van der Werf, G. R. *et al.* Global fire emissions and the contribution of deforestation, savanna, forest, agricultural, and peat fires (1997–2009). *Atmos Chem Phys* **10**, 11707-11735 (2010).
19. CarbonTracker CT2010, [http://carbontracker.noaa.gov](http://carbontracker.noaa.gov/) (2010).
20. Peters, W. *et al.* Seven years of recent European net terrestrial carbon dioxide exchange constrained by atmospheric observations. *Global Change Biol* **16**, 1317–1337 (2010).
21. GLOBALVIEW-CO2, Cooperative Atmospheric Data Integration Project - Carbon Dioxide. NOAA ESRL, Boulder, Colorado, available at [http://www.esrl.noaa.gov](http://www.esrl.noaa.gov/) (2010).
22. Masarie, K. A. & Tans, P. P. Extension and integration of atmospheric carbon dioxide data into a globally consistent measurement record. *J Geophys Res* **100**, 11593–11610 (1995).
23. Jiang, F. *et al.* Nested atmospheric inversion for the terrestrial carbon sources and sinks in China. *Biogeosciences* **10**, 5311-5324 (2013).
24. Liu, L. X. *et al.* The characteristics of atmospheric CO_2_ concentration variation of four national background stations in China. *Sci China Ser D-Earth Sci* **52**, 1857–1863 (2009).
25. Fang, S. X. *et al.* In situ measurement of atmospheric CO_2_ at the four WMO/GAW stations in China. *Atmos Chem Phys* **14**, 2541-2554 (2014).
26. Machida, T. *et al.* Worldwide measurements of atmospheric CO_2_ and other trace gas species using commercial airlines. *J Atmos Oceanic Technol* **25**, 1744–1754 (2008).
27. Matsueda, H. *et al.* Evaluation of atmospheric CO_2_ measurements from new flask air sampling of JAL airliner observations. *Pap Meteorol Geophys* **59**, 1–17 (2008).
28. Machida, T., Tohjima, Y., Katsumata, K. & Mukai, H. A new CO_2_ calibration scale based on gravimetric one-step dilution cylinders in National Institute for Environmental Studies -NIES 09 CO_2_ scale, GAW Rep. **194**, pp. 114–119, World Meteorol. Organ., Geneva, Switzerland (2011).
29. Zhang, H. F. *et al.* Estimating Asian terrestrial carbon fluxes from CONTRAIL aircraft and surface CO_2_ observations for the period 2006–2010. *Atmos Chem Phys* **14**, 5807-5824 (2014).
30. Niwa, Y. *et al.* Imposing strong constraints on tropical terrestrial CO_2_ fluxes using passenger aircraft based measurements. *J Geophys Res* **117**, D11303 (2012).
31. Bruhwiler, L. M. P., Michalak, A. M. & Tans, P. P. Spatial and temporal resolution of carbon flux estimates for 1983–2002. *Biogeosciences* **8**, 1309-1331 (2011).
32. Gurney, K. R. *et al.* Transcom 3 inversion intercomparison: model mean results for the estimation of seasonal carbon sources and sinks. *Global Biogeochem Cycles* **18**, GB1010 (2004).
33. Michalak, A. M. *et al.* Maximum likelihood estimation of covariance parameters for Bayesian atmospheric trace gas surface flux inversions. *J Geophys Res* **110**, D24107 (2005).
34. Rödenbeck, C. Estimating CO_2_ sources and sinks from atmospheric mixing ratio measurements using a global inversion of atmospheric transport, Technical Report 6, Max Planck Institute for Biogeochemistry, Jena (2005).
35. Chevallier, F. *et al.* CO_2_ surface fluxes at grid point scale estimated from a global 21 year reanalysis of atmospheric measurements. *J Geophys Res* **115**, D21307 (2010).
36. Le Quéré, C. *et al.* Global carbon budget 2013. *Earth Syst Sci Data* **6**, 235-263 (2014).
37. Boden, T. A., Marland, G. & Andres, R. J. Global, Regional, and National Fossil-Fuel CO_2_ Emissions. DOI 10.3334/CDIAC/00001_V2010 (2014).
38. Piao, S. L. *et al.* The carbon balance of terrestrial ecosystems in China. *Nature* **458**, 1009–1013 (2009).
39. Rayner, P. J. *et al.* Two decades of terrestrial carbon fluxes from a carbon cycle data assimilation system (CCDAS). *Global Biogeochem Cycles* **19**, GB2026 (2005).
40. Yu, G. R. *et al.* High carbon dioxide uptake by subtropical forest ecosystems in the East Asian monsoon region. *PNAS* **13**, 4910–4915 (2014).
41. Parton, W. J., Schimel, D. S., Cole, C. V. & Ojima, D. S. Analysis of factors controlling soil organic matter levels in Great Plains grasslands. *Soil Science Society of America Journal* **51**, 1173–1179 (1987).
42. Parton, W. J. *et al.* Observation and modeling of biomass and soil organic matter dynamics for the grassland and biome worldwide. *Global Biogeochemical Cycles* **7**, 785–809 (1993).
43. Farquhar, G. D., von Caemmerer, S. & Berry, J. A. A biochemical model of photosynthetic CO2 assimilation in leaves of C3 species. *Planta* **149**, 78–90 (1980).
44. Liu, J., Chen, J. M., Cihlar, J. & Chen, W. Net primary productivity distribution in the BOREAS study region from a process model driven by satellite and surface data. *Journal of Geophysical Research* **104**, 27735–27754 (1999).
45. Chen, W., Chen, J. & Cihlar, J. An integrated terrestrial ecosystem C-budget model based on changes in disturbance, climate, and atmospheric chemistry. *Ecological Modelling* **135**, 55–79 (2000).
46. Chen, J. M. *et al.* Spatial distribution of C sources and sinks in Canada’s forests based on remote sensing. *Tellus Series B: Chemical and Physical Meteorology* **55**, 622–641 (2003).
47. Liu, Y. *et al.* Changes of net primary productivity in China during recent 11 years detected using an ecological model driven by MODIS data. *Frontiers of Earth Science* **7**, 112 – 127 (2012).
48. Zhang, C. *et al.* Mapping forest stand age in China using remotely sensed forest height and observation data. *J Geophys Res Biogeosci* **119**, 1163–1179 (2014).
49. Shangguan, W. *et al.* A soil particle-size distribution dataset for regional land and climate modelling in China. *Geoderma* **171-172**, 85−91 (2012).
50. Wang, S. *et al.* Carbon sinks and sources in China's forests during 1901–2001. *Journal of Environmental Management* **85**, 524-537 (2007).
51. Shao, Y. *et al.* Tests of soil organic carbon density modeled by InTEC in China’s forest ecosystems. *Journal of Environmental Management* **85**, 696–701 (2007).
52. Zhang, Q. *et al.* Asian emissions in 2006 for the NASA INTEX-B mission. *Atmos Chem Phys* **9**, 5131-5153 (2009).
53. He, K. B. Multi-resolution Emission Inventory for China (MEIC): model framework and 1990–30 2010 anthropogenic emissions, International Global Atmospheric Chemistry Conference, 17–21 September, Beijing, China (2012).
54. Guenther, A. *et al.* Natural emissions of non-methane volatile organic compounds, carbon monoxide, and oxides of nitrogen from North America. *Atmospheric Environment* **34**, 2205-2230 (2000).
55. Klinger, L. F. *et al.* Assessment of volatile organic compound emissions from ecosystems of China. *J Geophys Res* **107**, 4603 (2002).
56. Yan, Y., Wang, Z. H., Bai, Y. H., Xie, S. D. & Shao, M. Establishment of vegetation VOC emission inventory in China. *China Environmental Science* **25**, 110-114 (2005). (in Chinese)
57. Xie, M., Wang, T. J., Jiang, F. & Yang, X. Q. Modeling of Natural NOx and VOC Emissions and Their Effects on Tropospheric Photochemistry in China. *Environmental Science* **28**, 32-40 (2007).
58. Kirschke, S. *et al.* Three decades of global methane sources and sinks. *Nature Geosci* **6**, 813-823 (2013).
59. Zheng, J. Y., Zheng, Z. Y., Yu, Y. F. & Zhong, L. J. Temporal, spatial characteristics and uncertainty of biogenic VOC emissions in the Pearl River Delta region, China. *Atmospheric Environment* **44**, 1960-1969 (2010).
60. Guan, D., Liu, Z., Geng, Y., Lindner, S. & Hubacek, K. The gigatonne gap in China's carbon dioxide inventories. *Nature Clim* *Change* **2**, 672–675 (2012).
61. Ciais, P. *et al.* The impact of lateral carbon fluxes on the European carbon balance. *Biogeosciences* **5**, 1259–1271 (2008).
62. Zhai, W. D., Dai, M. H. & Guo, X. G. Carbonate system and CO_2_ degassing fluxes in the inner estuary of Changjiang (Yangtze) River, China. *Marine Chemistry* **107**, 342-356 (2007).
63. Ran, L. S. *et al.* Spatial and seasonal variability of organic carbon transport in the Yellow River, China. *Journal of Hydrology* **498**, 76-88 (2013).
64. Wu, Y. *et al.* Sources and distribution of carbon within the Yangtze River system. *Estuar Coast Shelf Sci* **71**, 13–25 (2007).
65. Xia, B. & Zhang, L. J. Carbon distribution and fluxes of 16 rivers discharging into the Bohai Sea in summer. *Acta Oceanologica Sinica* **30**, 43-54 (2011).
66. Wang, X., Ma, H., Li, R., Song, Z. & Wu, J. Seasonal fluxes and source variation of organic carbon transported by two major Chinese Rivers: The Yellow River and Changjiang (Yangtze) River. *Global Biogeochem Cycles* **26**, GB2025 (2012).
67. Ludwig, W., Amiotte-Suchet, P., Munhoven, G. & Probst, J. L. Atmospheric CO_2_ consumption by continental erosion: present-day controls and implications for the last glacial maximum. *Global Planet Change* **17**, 107–120 (1998).
68. Ludwig, W., Probst, J. L. & Kempe, S. Predicting the oceanic input of organic carbon by continental erosion. *Global Biogeochemical Cycles* **10**, 23-41 (1996).
69. Zhu, X. J., Yu, G. R., Gao, Y. N. & Wang, Q. F. Fluxes of Particulate Carbon from Rivers to the Ocean and Their Changing Tendency in China. *Progress in Geography* **31**, 118 – 122 (2012).
70. The Ministry of Water Resources of the People's Republic of China. China's river sediment bulletin 2006. Beijing: China WaterPower Press (2006).
71. The Ministry of Water Resources of the People's Republic of China. China's river sediment bulletin 2007. Beijing: China WaterPower Press (2007).
72. The Ministry of Water Resources of the People's Republic of China. China's river sediment bulletin 2008. Beijing: China WaterPower Press (2008).
73. The Ministry of Water Resources of the People's Republic of China. China's river sediment bulletin 2009. Beijing: China WaterPower Press (2009).
74. Yao, G. R. *et al.* Dynamics of CO_2_ partial pressure and CO_2_ outgassing in the lower reaches of the Xijiang River, a subtropical monsoon river in China. *Science of the Total Environment* **376**, 255-266 (2007).
75. Wang, F. S., Wang, Y. C., Zhang, J., Xu, H. & Wei, X. G. Human impact on the historical change of CO_2_ degassing flux in River Changjiang. *Geochemical Transactions* **8**, 7 (2007).
76. Li, S. *et al.* Daily CO_2_ partial pressure and CO_2_ outgassing in the upper Yangtze River basin: A case study of the Longchuan River, China. *Journal of Hydrology* **466-467**, 141-150 (2012).
77. Wang, F. S. *et al.* Carbon dioxide emission from surface water in cascade reservoirs-river system on the Maotiao River, southwest of China. *Atmospheric Environment* **45**, 3827-3834 (2011).
78. Butman, D. & Raymond, P. A. Significant efflux of carbon dioxide from streams and rivers in the United States. *Nature Geoscience* **4**, 839–842 (2011).
79. National Bureau of Statistics of the People's Republic of China. China Statistical Yearbook, <http://www.stats.gov.cn/tjsj/ndsj/2008/indexeh.htm> (2008).
80. Tranvik, L. J. *et al.* Lakes and reservoirs as regulators of carbon cycling and climate. *Limnol Oceanogr* **54**, 2298–2314 (2009).
81. Yang, L. *et al.* Spatial and seasonal variability of CO_2_ flux at the air-water interface of the Three Gorges Reservoir. *Journal of Environmental Sciences* **25**, 2229-2238 (2013).
82. St Louis, V. L., Kelly, C. A., Duchemin, É., Rudd, J. W. M. & Rosenberg, D. M. Reservoir surfaces as sources of greenhouse gases to the atmosphere: a global estimate. *Bioscience* **50**, 766-775 (2000).
83. Gui, Z. F., Xue, B., Yao, S. C., Wei, W. J. & Yi, S. Organic carbon burial in lake sediments in the middle and lower reaches of the Yangtze River Basin, China. *Hydrobiologia* **710**, 143-156 (2013).
84. Dong, X., Anderson, N. J., Yang, X., Chen, X. & Shen, J. Carbon burial by shallow lakes on the Yangtze floodplain and its relevance to regional carbon sequestration. *Global Change Biology* **18**, 2205–2217 (2012).
85. Cole, J. J. *et al.* Plumbing the global carbon cycle: Integrating inland waters into the terrestrial carbon budget. *Ecosystems* **10**, 171–184 (2007).
86. FAO Statistical databases. Available at http:// faostat3.fao.org (2013).
87. Winjum, J. K., Brown, S. & Schlamadinger, B. Forest harvests and wood products: sources and sinks of atmospheric carbon dioxide. *Forest Science* **44**, 272-284 (1998).
88. Pan, Y. *et al.* A large and persistent carbon sink in the world’s forests. *Science* **333**, 988–993 (2011).
89. Zhang, L. K., Qin, X. Q., Yang, H., Huang, Q. B. & Liu, P. Y. Transported Fluxes of the Riverine Carbon and Seasonal Variation in Pearl River Basin. *Environmental Science* **34**, 3025-3034 (2013).
